# Clinical Outcomes of *Campylobacter* Bacteremia: A Systematic Review with Meta-Analysis

**DOI:** 10.3390/pathogens15070686

**Published:** 2026-06-29

**Authors:** Verena Zerbato, Stefano Guicciardi, Roberto Baldan, Chiara Fanelli, Lisa Fusaro, Alexandru Botan, Nicola Benvenuto, Giovanna Maria Nicolò, Luigi Principe, Dan Alexandru Toc, Mauro Giuffrè, Lory Saveria Crocé, Luca Frulloni, Abbas Yadegar, Stefano Di Bella, Alberto Enrico Maraolo

**Affiliations:** 1 Infectious Diseases Unit, Trieste University Hospital (Azienda Sanitaria Universitaria Giuliano Isontina), 34125 Trieste, Italy; 2 Health Directorate, Local Health Authority of Bologna, 40124 Bologna, Italy; 3 Department of Biomedical and Neuromotor Sciences, University of Bologna, 40138 Bologna, Italy; 4 Department of Medicine, University of Verona, 37124 Verona, Italy; 5 Department of Medicine, Surgery and Pharmacy, University of Sassari, 07100 Sassari, Italy; 6 Clinical Department of Medical, Surgical and Health Sciences, Trieste University, 34129 Trieste, Italy; 7 Department of Microbiology, "Iuliu Hațieganu" University of Medicine and Pharmacy, 400012 Cluj-Napoca, Romania; 8 Microbiology and Virology Unit, Great Metropolitan Hospital “Bianchi-Melacrino-Morelli”, 89100 Reggio Calabria, Italy; 9 Department of Internal Medicine (Digestive Diseases), Yale School of Medicine, Yale University, New Haven, CT 06510, USA; 10 Liver Clinic, Trieste University Hospital (Azienda Sanitaria Universitaria Giuliano Isontina), 34125 Trieste, Italy; 11 Foodborne and Waterborne Diseases Research Center, Research Institute for Gastroenterology and Liver Diseases, Shahid Beheshti University of Medical Sciences, Tehran 1985717411, Iran; 12 Department of Clinical Medicine and Surgery, Section of Infectious Diseases, University of Naples ’Federico II’, 80131 Napoli, Italy

**Keywords:** *Campylobacter* spp., bacteremia, bloodstream infection, meta-analysis, systematic review, One Health

## Abstract

Purpose: *Campylobacter* spp. are a common cause of acute enteric infections. In immunocompromised or elderly patients, they can lead to extraintestinal infections, including bacteremia. The clinical significance of *Campylobacter* bacteremia is not fully understood. Methods: We conducted a systematic review and meta-analysis on *Campylobacter* spp. bacteremia, including studies published up to June 2024. Results: Twenty-five retrospective observational studies, published between 1978 and 2024, were included. The studies involved a total of 2480 patients, with a mean age range across studies from 1 to 70 years; 62.45% were male. The pooled prevalence of *Campylobacter* species was: *C. jejuni* 60% [95% CI 0.45–0.73], *C. coli* 8% [95% CI 0.04–0.13], *C. fetus* 7% [95% CI 0.03–0.15], and other species 9% [95% CI 0.04–0.16]. Mortality was the primary outcome in 22 studies, with a pooled case-fatality risk of 5% [95% CI 0.03–0.09]. Univariate meta-regression showed higher mortality associated with *C. fetus* (β = 3.217, [95% CI 0.632–5.802], *p* = 0.017), immunocompromised status (β = 2.749, [95% CI 0.316–5.184], *p* = 0.029), and chronic liver disease (β = 5.072, [95% CI 0.424–9.720], *p* = 0.034). Regarding complications, secondary localizations (e.g., endovascular infections) showed a pooled prevalence of 9% [95% CI: 0.04–0.18]; relapses, 3% [95% CI 0.02–0.04]; endocarditis, 2% [95% CI 0.01–0.03]; and persistent bacteremia, 1% [95% CI 0.001–0.27]. Conclusion: *Campylobacter* spp. bacteremia shows a considerable risk of mortality and complications.

## 1. Introduction

*Campylobacter* spp. are a common cause of acute enteric infections in humans [1]. In Europe, in 2024, there were 168,396 confirmed cases of human campylobacteriosis, corresponding to a notification rate of 55.3 cases per 100,000 of the population, showing an increase compared to 2023 [2]. *Campylobacter jejuni* is the most significant species and the leading cause of gastroenteritis in humans worldwide. It is followed by *Campylobacter coli* and *Campylobacter fetus*. Other *Campylobacter* species, such as *Campylobacter hyointestinalis*, *Campylobacter upsaliensis*, *Campylobacter lari*, and *Campylobacter ureolyticus*, are emerging as causes of human infections [3]. Poultry is considered the principal reservoir of *Campylobacter* spp., but cattle, domestic animals and swine can also be involved. Human infection typically results from ingesting contaminated food, milk or water [4]. Other well-known risk factors are international travel and direct contact with farm animals [5].

The incubation period of campylobacteriosis ranges from two to five days after exposure [1]. Clinical presentations include abdominal pain, fever, nausea, and/or vomiting [1]. Although being mostly self-limiting, lasting around a week, hospitalization is required in up to 23% of cases in Europe [2]. Among immunocompromised hosts, elderly people, and children, *Campylobacter* spp. can cause serious extraintestinal infections, especially bacteremia [6,7]. In this context, *C. fetus* stands out among *Campylobacter* species as being more frequently associated with invasive infections [8].

To date, no specific international guidelines are available for campylobacteriosis management and treatment. Nonetheless, the most used antibiotics include fluoroquinolones, macrolides, and tetracyclines. Infections caused by *C. fetus* are typically managed with parenteral antibiotic therapy, particularly aminoglycosides and/or carbapenems [9]. Antimicrobial resistance in *Campylobacter* spp. is increasing worldwide [10], and reports of multidrug-resistant (MDR) strains, particularly *C. coli*, are becoming increasingly common [11]. In 2022 the European Centre for Disease Prevention and Control (ECDC) reported high resistance to fluoroquinolones (69.1 % for *C. jejuni* and 70.6 % for *C. coli*) and tetracycline (46.6 % for *C. jejuni* and 71.2 % for *C. coli*). Macrolides still retain good activity against *Campylobacter* spp. (0.9 % resistance for *C. jejuni* and 7.8 % for *C. coli*) [12].

Bloodstream infections (BSIs) are generally correlated to augmented mortality [13]. However, the clinical relevance of *Campylobacter* spp. bacteremia remains poorly defined, particularly with regard to species-specific prognosis, complications, predictors of mortality, and the role of appropriate antimicrobial therapy. Therefore, we conducted a systematic review and meta-analysis to assess the prevalence, clinical characteristics, and outcomes of *Campylobacter*-associated bacteremia.

## 2. Methods

This study followed the Preferred Reporting Items for Systematic Reviews and Meta-Analyses (PRISMA) guidelines [14]. The study protocol was registered with PROSPERO (registration number: CRD42024504893) on 1 February 2024.

### 2.1. Data Sources and Search Strategy 

For this systematic review and meta-analysis, we searched MEDLINE, Embase, and the Web of Science Database using specific keywords and terms related to *Campylobacter* bacteremia.

The searches covered studies in humans published from inception until December 31, 2023. The search was re-run to update the data collection until June 2024. 

The complete research strategy is reported in the Supplementary Materials.

Six reviewers (SG, VZ, AB, CF, LF, RB) independently screened the titles and abstracts of articles identified through the electronic searches against the eligibility criteria. The full texts of potentially relevant articles were then independently assessed, with discrepancies resolved by consensus among the entire study group. 

### 2.2. Study Selection

Eligible studies included observational studies (both retrospective and prospective) and case series with at least 15 patients, examining patients of all ages diagnosed with *Campylobacter* BSI. The studies had to report at least one of the two primary outcomes. We included papers published in English, without geographical restrictions. 

### 2.3. Data Extraction

Data was extracted into a standardized report, documenting the following: first author, year of publication, country, study design, baseline characteristics (median age, gender distribution, main comorbidities), setting, number of patients, *Campylobacter* species involved, risk factors (e.g., consumption of undercooked meat, recent travel, contact with livestock, recent abdominal surgery or trauma, outbreak involvement), identification method, resistance rates (e.g., to ciprofloxacin, erythromycin, tetracycline, gentamicin, and meropenem), clinical presentation (e.g., fever, sepsis, septic shock, gastrointestinal symptoms, coinfections), treatment (drug and duration), and outcomes (30-day mortality and complications). Data extraction was conducted by six reviewers (SG, VZ, AB, CF, LF, RB).

Discrepancies in data extraction were resolved by discussion. If adjusted effect sizes related to mortality predictors were available from multivariable models, these were also extracted.

Attempts were made to contact authors via email to obtain relevant information not available in the full texts.

### 2.4. Assessment of Study Bias

Quality assessment of the included studies was independently carried out through the tool developed by Hoy and colleagues [15] by four reviewers (VZ, RB, CF and LF). Discrepancies were resolved by consensus among the entire study group. A score of 1 (yes) or 0 (no) was attributed to each item, producing a final quality score range from 0 to 10 (poor to good).

### 2.5. Study Outcome Definition

Our study had two co-primary outcomes. The first was to assess the pooled, relative prevalence of different *Campylobacter* spp. strains responsible for BSIs globally, mainly (but not only) *C. jejuni*, *C. fetus,* and *C. coli*. The second was to assess the overall case-fatality risk associated with *Campylobacter* spp. BSIs.

The secondary outcomes were the following: to evaluate the main antimicrobial resistance patterns among *Campylobacter* spp. strains responsible for BSIs; to determine the most frequent clinical manifestations accompanying *Campylobacter* spp. BSIs; and to assess the proportions of the most relevant complications of *Campylobacter* spp. BSIs. Moreover, a comparison between cases undergoing appropriate treatment (defined as the receipt of at least one active antimicrobial agent in vitro) and the ones not receiving appropriate treatment with all-cause mortality as an outcome was performed.

Pre-planned sensitivity analyses were carried out to verify the robustness of the results in the face of potential outliers.

The impact of pre-specified covariates on the relative proportion of the different *Campylobacter* species and on the case-fatality risk due to *Campylobacter* spp. BSIs was assessed. For instance, how the proportion of a given species and how the burden of death varied over time; the impact of time as moderator was also analyzed for resistance to key antibiotics.

Although the original study protocol included, as additional outcomes, the assessment of the main risk factors for *Campylobacter* spp. BSIs and the most frequently prescribed antimicrobial regimens, the data ultimately proved too limited to allow for a proper analysis.

### 2.6. Statistical Analysis

In the light of the anticipated large variation in the regions and times of the included studies, we resorted to a random-effects model for the meta-analysis. The prevalence of each species was calculated by dividing the number of relative cases by the overall number of BSIs by *Campylobacter* spp. in each study, and the case-fatality risk was computed similarly. In line with the newest development regarding meta-analysis of proportions, to calculate the pooled prevalences of interest with 95% confidence intervals (CIs) we used generalized linear mixed models (GLMMs), a one-step approach allowing us to fully account for within-study uncertainties, implying smaller biases and mean squared errors, and higher coverage probabilities than two-step methods [16]. Among the various methods available to CIs for individual study results, the Clopper–Pearson interval was chosen. The restricted maximum likelihood estimator [17] was used to calculate the heterogeneity variance τ^2^. We used Knapp–Hartung adjustments [18] to compute the CI around the pooled effect.

Regarding the comparison between appropriate versus inappropriate treatment, in anticipation of a meta-analysis of rare events, the analysis itself was conducted within a proposed framework to guide evidence synthesis, particularly in cases involving studies with zero-events in one or both arms [19]. Our case fell into a category defined as “MA-MZ” within this framework: zero-events occurring in both single and double arms, and the total events count in neither arm is zero; pooled odds ratios (ORs) with 95% CIs were also estimated in this case, resorting to a GLMM with an exact noncentral hypergeometric-normal likelihood [19]. This one-stage approach treats each study as a stratum or cluster, allowing the overall effect size to be computed using the population average method [19]. When appropriate, risk difference (RD) was used as an alternative to OR, following the guidelines of the aforementioned framework [19].

Statistical heterogeneity between studies was evaluated using Cochran’s Q statistic and the I^2^ statistic, with heterogeneity classified as low, moderate, or high for I^2^ values of less than 50%, 50–74%, and 75% or greater, respectively [20].

Alongside pooled effect sizes with their 95% CIs, a prediction interval was also reported to account for the variation in treatment effects across different settings, including potential effects in future patients [21].

Additionally, meta-regression was performed to explore between-study heterogeneity, considering several pre-specified study-level variables based on clinical plausibility. The meta-regression yielded regression coefficients showing how the pooled prevalence varied across categories of categorical factors or increases with a unit increase in continuous explanatory variables such age or the proportion of patients with given features (e.g., percentage of immunosuppressed, subjects with diabetes mellitus and so on). Regarding the first co-primary outcome (relative proportion of *Campylobacter* spp. strains responsible for BSIs), a univariate meta-regression considering the mid-year of each observation as a moderator effect was carried out. Concerning the second co-primary outcome (case-fatality risk due to *Campylobacter* spp. BSIs), a univariate meta-regression involving several potential moderators was performed: predictors that were found to be related to the outcome (*p* value ≤ 0.20) were then entered in a multiple meta-regression model wherein the threshold for statistical significance was set at *p* ≤ 0.05. 

For continuous variables, means and standard deviations (SDs) were obtained from sample size, medians, interquartile ranges (IQRs), and minimum/maximum values if not provided [22].

In case of missing values among predictors, data were imputed through multiple imputation by chained equations assuming that data were missing at random [23].

A subgroup analysis was conducted only based on geographical area, defined according to the World Health Organization regional grouping of countries.

Additionally, sensitivity analyses about the two co-primary outcomes were performed to assess the impact on effect size and I^2^ by removing one study at a time (leave-one-out analysis).

Eventually, Doi plots and the Luis Furuya–Kanamori (LFK) index were used to assess if small-study effects were present, to assess publication bias. The LFK index values on a symmetrical mountain-like graph fall into three categories: within ±1, no asymmetry is indicated; between ±1 and ±2, moderate asymmetry is indicated; and exceeding ±2 shows substantial asymmetry [24].

All analyses were performed using R (R language; R Foundation for Statistical Computing, Vienna, Austria), exploiting the following packages: *dmetar*, *meta*, *metafor*, *mice*. Only the Doi plots with the related LFK index were obtained by means of MetaXL version 5.3 (Ersatz, EpiGear International, Sunrise Beach, Australia) [25].

### 2.7. Deviations from Protocol

Two deviations from the pre-registered protocol occurred during the review process. First, we included studies reporting at least 15 cases of *Campylobacter* spp. bacteremia, whereas the original protocol specified a minimum of 20 cases. This change was made to increase the number of eligible studies and better reflect the rarity of the condition. The revised threshold was based only on sample size and was applied uniformly, independently of study results or reported outcomes, thereby limiting the risk of meaningful selection bias. Second, while the protocol initially planned for no language restrictions, the inclusion criteria were revised to include only studies published in English due to resource constraints. These deviations are acknowledged to ensure transparency and reproducibility.

## 3. Results

### 3.1. Systematic-Review Search Results 

**Figure 1:**
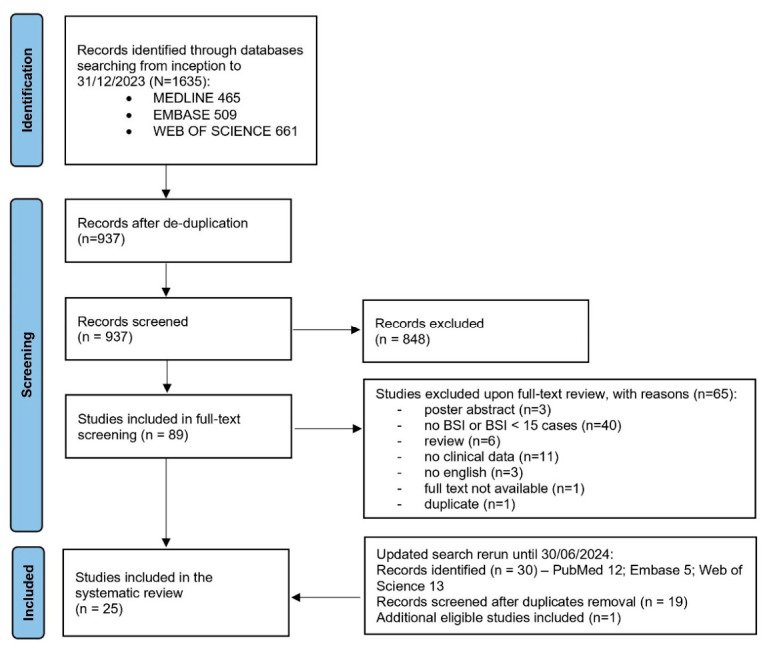
Literature selection procedure.

Through the search, 509 studies were obtained from Embase, 661 from Web of Science and 465 from Medline. After removal of duplicates, 937 studies were screened, out of which 89 full-text articles were assessed for eligibility. Sixty-five were excluded, leaving a total 24 studies to be included. Another eligible study identified after the search was re-run to update data collection until June 2024 has been included. The PRISMA diagram showing the flow of study selection, including the identification, screening, eligibility, and inclusion of studies, is presented in Figure 1. 

### 3.2. Study Characteristics and Methodological Assessment

No randomized controlled trials were found during the search period. All studies included in this review were case series and retrospective observational cohort studies, published between 1978 and 2024. The geographical distribution of the studies was as follows: 12 from Europe, 7 from Asia, 3 from Australia, 2 from Africa, and one from America. Quality assessment of the included studies was carried out through the tool developed by Hoy and colleagues [15]. A score of 1 (yes) or 0 (no) was attributed for each item, producing a final quality score range from 0 to 10 (poor to good), as shown in Supplementary Table S1. Among the included studies, the risk of bias was assessed as moderate in ten cases; in all remaining studies, it was judged to be low.

The study characteristics are summarized in Table 1.

### 3.3. Patients’ Characteristics in Meta-Analyses

The 25 papers included in the meta-analysis reported a total of 2480 patients, with a mean age widely ranging across studies, the lowest equal to 1 year [6] and highest equal to 70 years [35]. Overall, 62.45% of the patients were male. All studies except one reported the comorbidities of the included patients. Only nine studies reported risk factors for BSI. Among them, the most frequently reported was contact with livestock/cattle (in 48 patients), followed by recent travel (in 24 patients). For further details on patient characteristics, see Table 1.

### 3.4. Prevalence Meta-Analyses

**Figure 2:**
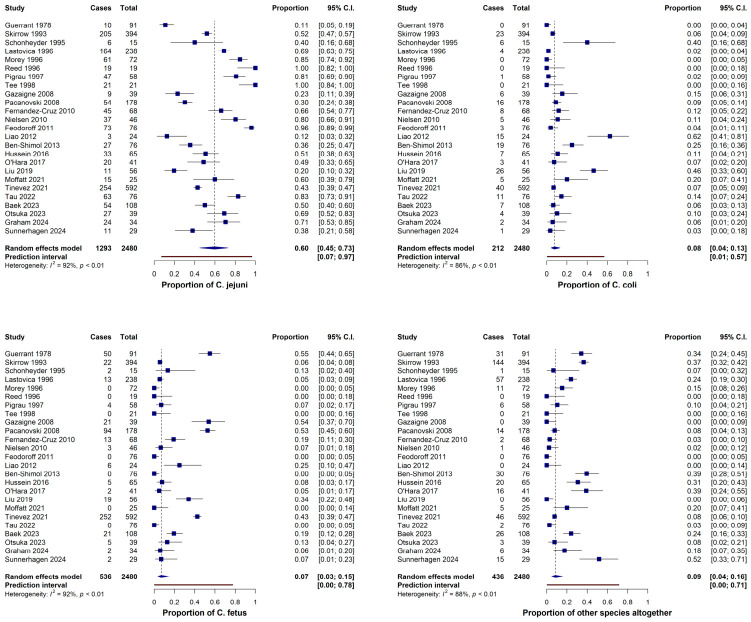
Forest plots illustrating the pooled prevalence of *Campylobacter* species: *C. jejuni*, *C. coli*, *C. fetus*, and other species.

The pooled prevalences worldwide of *Campylobacter* species have been reported as follows: *C. jejuni* was 60% [95% CI 0.45–0.73], *C. coli* was 8% [95% CI 0.04–0.13], *C. fetus* was 7% [95% CI 0.03–0.15], and other species was 9% [95% CI 0.04–0.16], as shown in Figure 2. 

### 3.5. Mortality Meta-Analyses

**Figure 3:**
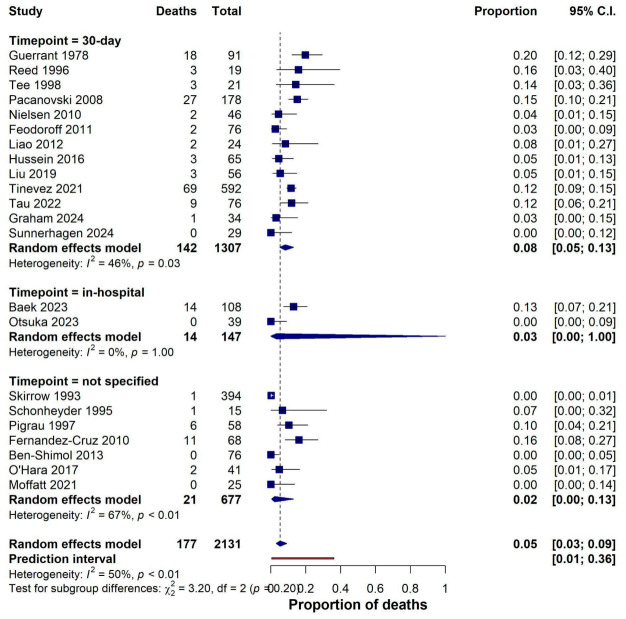
Forest plot illustrating the pooled analysis of mortality outcome.

Twenty-two studies reported mortality as a main outcome, with 13 specifying 30 days from the diagnosis of bacteremia as the time point, 2 reporting in-hospital mortality, and 7 not specifying the time point. The overall case-fatality risk associated with *Campylobacter* spp. BSI was 5% [95% CI 0.03–0.09] (Figure 3). Supplementary Figure S18 presents a forest plot of the pooled mortality analysis stratified by the World Health Organization region.

### 3.6. Secondary Outcomes

We evaluated the main antimicrobial resistance patterns among *Campylobacter* spp. strains responsible for BSIs. The pooled prevalence of strains resistant to ciprofloxacin, erythromycin, and tetracycline were as follows: 37% [95% CI 0.25–0.50], 4% [95% CI 0.03–0.06], and 12% [95% CI 0.02–0.51] (Supplementary Figures S1–S3). Resistance to gentamicin and carbapenem were also investigated and the pooled prevalences were 1% [95% 0.00–0.05] and 0% [95% 0.00–0.32], respectively (Supplementary Figures S4 and S5). Resistance to two or three classes of antibiotics simultaneously was not reported.

Regarding clinical presentation, the pooled prevalence of patients with fever was 75% [95% CI 0.63–0.85], while 61% [95% CI 0.52–0.70] presented with gastrointestinal symptoms. Additionally, 34% of patients with *Campylobacter* BSI had a concomitant positive stool culture [95% CI 0.15–0.59] (Supplementary Figures S6–S8). 

We further analyzed complications associated with *Campylobacter* BSI. The pooled prevalence of secondary localization in *Campylobacter* bacteremia was 9% [95% CI 0.04–0.18], while the pooled prevalence of endocarditis was 2% [95% CI 0.01–0.03] (Supplementary Figures S9 and S10). Other common secondary localizations were endovascular infection, skin and soft tissue infections, and osteomyelitis. The pooled prevalence of relapses was 3% [95% CI 0.02–0.04], while it was 1% for persistent BSI [95% CI 0.001–0.27] (Supplementary Figures S11 and S12).

**Figure 4:**
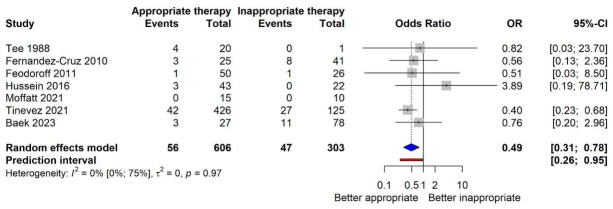
Forest plot of studies comparing appropriate versus inappropriate therapy.

Pooled analysis demonstrated that appropriate therapy was significantly associated with a reduced risk of mortality compared to inappropriate therapy (OR 0.49, 95% CI 0.31–0.78; prediction interval 0.26–0.95), with no observed heterogeneity (I^2^ = 0%) (Figure 4).

### 3.7. Meta-Regression

Univariate meta-regression showed no statistically significant temporal trends in the global proportions of *Campylobacter* species. The coefficient for *C. coli* was 0.046 (95% CI, −0.003–0.096, *p* = 0.066), suggesting a possible increasing trend. All other species showed non-significant associations with time (Table 2).

**Figure 5:**
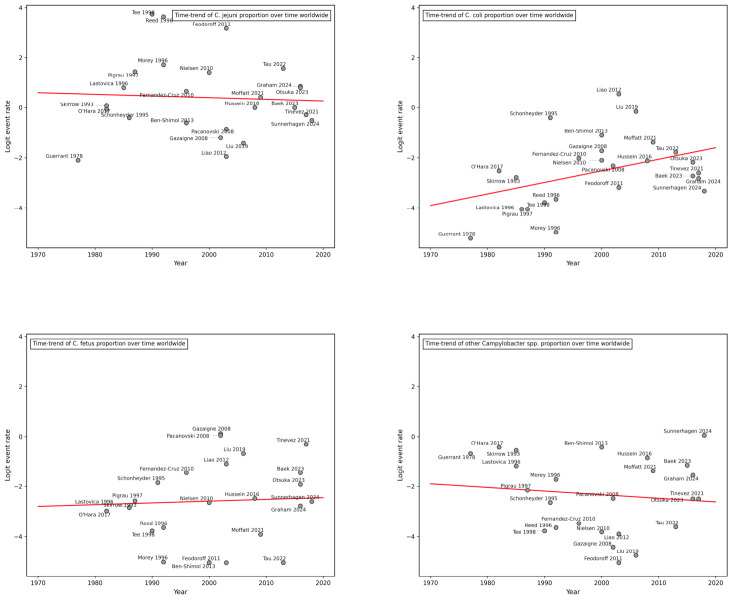
Time trends in the proportion of *Campylobacter* species worldwide based on univariate meta-regression. Each plot shows logit event rates by year with fitted regression lines for *C. jejuni*, *C. coli*, *C. fetus*, and other species. The red line in each panel is the fitted regression line from the univariate meta-regression, showing the predicted logit-transformed proportion of each *Campylobacter* group as a function of study year. The slope represents the estimated change in the logit proportion per calendar year: a downward-sloping line indicates a declining proportion over time, and an upward-sloping line an increasing proportion.

These findings are visually reflected in the scatterplots and fitted regression lines shown in Figure 5.

Univariate meta-regression on death found out that *C. fetus* (β = 3.217, [95% CI 0.632–5.802], *p* = 0.017) immunocompromised status (β = 2.749, [95% CI 0.316–5.184], *p* = 0.029), and chronic liver disease (β = 5.072, [95% CI 0.424–9.720], *p* = 0.034) are associated with an increased risk of mortality in patients with *Campylobacter* bacteremia. Interestingly, infections caused by *Campylobacter* species other than *C. jejuni*, *C. coli*, and *C. fetus* were associated with a significantly lower risk of death (β = -3.808, 95% CI -7.503 to -0.114; *p* = 0.044) (Table 3). 

However, in the multivariate meta-regression model, none of these variables remained statistically significant. *C. fetus* and other *Campylobacter* species showed borderline associations with mortality (*p* = 0.061 and *p* = 0.065, respectively), while the effects of immunocompromised status and chronic liver disease were attenuated (Table 4). 

Additional meta-regression analyses on antimicrobial resistance trends are available in the Supplementary Material (Supplementary Figure S14 and S15, Table S5).

## 4. Discussion

The findings from this systematic review and meta-analysis highlight that *Campylobacter* spp. bacteremia, although relatively rare compared to enteric infections, is associated with a non-negligible mortality rate and a considerable risk of complications. The pooled case-fatality rate was 5% (95% CI 0.03–0.09). 

Among the *Campylobacter* species, *C. jejuni* accounted for most blood isolates (pooled worldwide prevalence of 60%), whereas *C. fetus* represented 7%. Although less frequent, *C. fetus* infection was significantly associated with an increased risk of mortality in univariate meta-regression analysis (β = 3.217, *p* = 0.017); however, this association was attenuated in the multivariate model. Some previous studies even suggested that *C. fetus* causes a disproportionately higher rate of invasive infections compared to other *Campylobacter* species, underscoring its propensity for systemic spread [11]. Nevertheless, the specific pathogenic mechanisms that differentiate *C. fetus* from other *Campylobacter* spp. remain poorly understood. Two major virulence factors have been identified that may explain the heightened pathogenicity of *C. fetus*: a type IV secretion system and surface layer proteins, which undergo antigenic variation and shield the bacterium from host immune defenses, facilitating immune evasion [47,48]. 

In terms of complications, secondary localization was observed in 9% of patients (95% CI 0.04–0.18), while endocarditis occurred in 2% (95% CI 0.01–0.03). Other common secondary localizations included endovascular infections, skin and soft tissue infections, and osteomyelitis [7,8,38,49]. In the available studies, *C. fetus* was the species most frequently associated with secondary localizations. These findings highlight the need for clinicians to actively consider and investigate potential secondary localizations in patients with *Campylobacter* bacteremia, especially in those presenting with persistent symptoms or risk factors for metastatic infection. However, these estimates should be interpreted with caution, as complication data were available from only 13 studies and reporting was uneven across cohorts. Notably, a concomitant positive stool culture was present in only 34% of cases, highlighting that bacteremia can occur in the absence of ongoing enteritis symptoms—a finding that has diagnostic implications.

Host-related factors were equally important. In univariate meta-regression analyses, immunocompromised status was associated with an increased risk of death (β = 2.749, *p* = 0.029), as was chronic liver disease (β = 5.072, *p* = 0.034), although these associations were attenuated in the multivariable model. This is not surprising, as most *Campylobacter* bacteremia cases reported in the literature have occurred in these high-risk populations [7,26]. Regarding immunosuppression, the wide heterogeneity of reported conditions—ranging from solid organ transplantation to hematologic malignancies and congenital immunodeficiencies—precluded any meaningful stratified analysis.

Antibiotic therapy played a crucial role in patient outcomes. Our pooled analysis demonstrated that appropriate therapy was significantly associated with reduced mortality (OR 0.49, 95% CI 0.31–0.78). However, this analysis was based on only seven studies, and due to the limited number of events, it was not possible to perform any stratified analysis according to the specific type of antibiotic regimen. Although there are no standardized international guidelines for the treatment of *Campylobacter* BSIs, current practices favor fluoroquinolones, macrolides, or tetracyclines for mild enteric disease. However, for systemic infections, particularly those caused by *C. fetus*, parenteral agents such as carbapenems or aminoglycosides are often necessary [9]. Resistance patterns support this approach: the pooled resistance rates were 37% for ciprofloxacin and 12% for tetracyclines, but only 4% for macrolides, and virtually 0–1% for gentamicin and carbapenems. According to global surveillance data on *Campylobacter* resistance, these numbers are quite low [12,50]. This likely reflects the broad time span of the studies included in the review. 

The overall quality of the evidence included in this review can be considered moderate. All 25 studies were observational in design, predominantly retrospective, and no randomized controlled trials were identified. This may limit the ability to draw causal inferences, although the overall risk of bias was low in the vast majority of studies. Recently, a systematic review and meta-analysis on *Campylobacter* bacteremia including fewer cases reported a mortality rate of 7.2%, consistent with our findings and further underscoring their clinical relevance [51]. 

The study has some limitations. Firstly, only English-language studies were included, which could have introduced selection bias. Secondly, since most studies originated from Europe, the geographic imbalance may have contributed to publication bias. Thirdly, the studies span 1978 to 2024, a period marked by substantial changes in diagnostic technologies, therapeutic strategies, population health characteristics, and the taxonomy of *Campylobacter* spp. Finally, differences in diagnostic protocols, microbiological methods, and definitions of outcomes may have introduced heterogeneity; therefore, some pooled estimates should be interpreted as context-dependent rather than universally generalizable.

Future research should aim to address these gaps. Prospective multicenter studies would help clarify optimal treatment strategies, including duration and choice of antibiotics for different *Campylobacter* species. Given the particularly severe course of *C. fetus* infections, longer or combination therapies may be warranted, but data are currently insufficient. Furthermore, exploration of host–pathogen interactions and *Campylobacter* virulence factors may provide insight into mechanisms of invasive disease and identify potential therapeutic targets.

In conclusion, *Campylobacter* bacteremia is a clinically relevant condition associated with non-negligible mortality and a substantial risk of complications. While outcomes are generally favorable, *C. fetus* infection and immunosuppression may identify patients at increased risk of death. Prompt detection, species-level identification, susceptibility testing, and appropriate antibiotic therapy are essential to optimize patient outcomes.

## Figures and Tables

**Table 1:** Included studies.

Author (Country, Year)	Study design and period	N° of *Campylobacter* BSIs (*jejuni; coli; fetus*; others)	Mean age (years)	N° of male/all subjects (%)	Comorbidities (n°)	Setting	Risk factors (n°)	N° of patients with concomitant positive stool culture	Antibiotics resistance (n° of resistant strains)	Signs and symptoms at BSI diagnosis	Treatment (drugs, combination, days of therapy, appropriate therapy)	Mortality (time point)	Complications	Reference
Skirrow M.B. (UK, 1993)	Retrospective multicentric (1981-1991)	394 (205; 23; 22; 144)	45.9	165/250 (66%)	Median CCI n/a DM 11 CRF 16 CLD 15 CVD n/a PHV n/a IC 74	n/a	n/a	n/a	n/a	Fever n/a Sepsis n/a Septic shock n/a GI symptoms 170 Coinfections n/a	n/a	1/394 (time point not specified)	n/a	[26]
Schønheyder H.C. (Denmark, 1995)	Retrospective multicentric (1989-1994)	15 (6; 6; 2; 1)	49	7/15 (46.7%)	Median CCI n/a DM 2 CRF 1 CLD 2 CVD 2 PHV 1 IC 6	n/a	2 undecooked meat 2 recent travel 1 contact with livestock/cattle	8	CIP 0/15 ERY 2/15 TET 0/15 GEN 0/15 MEM n/a	Fever 14 Sepsis n/a Septic shock n/a GI symptoms 8 Coinfections n/a	Combination therapy (n°) 6	1/15 (time point not specified)	Relapses 2/15 IE 1/25 Persistent BSI n/a Secondary localizations n/a	[27]
Pigrau C. (Spain, 1997)	Retrospective monocentric (1979-1996)	58 (47; 1; 4; 6)	39.4	38/58 (65.5%)	Median CCI n/a DM 4 CRF 3 CLD 20 CVD n/a PHV n/a IC 13	n/a	n/a	15	CIP 13/24 ERY 4/58 TET 0/58 GEN 0/58 MEM n/a	Fever 53 Sepsis n/a Septic shock 1 GI symptoms 18 Coinfections n/a	N° days of therapy (range) 14-21	6/57 (time point not specified)	Relapses 1/57 IE n/a Persistent BSI n/a Secondary localizations 11/57	[28]
Pacanowski J. (France, 2008)	Retrospective multicentric (2000-2004)	178 (54;16; 94; 14)	64	124/178 (69.7%)	Median CCI n/a DM 31 CRF n/a CLD 69 CVD n/a PHV n/a IC 141	n/a	n/a	13	CIP 50/157 ERY 12/156 TET n/a GEN 4/147 IPM 0/73	Fever 74 Sepsis n/a Septic shock n/a GI symptoms 58 Coinfections 7	IPM (n°) 12	27/178 (30-day)	Relapses n/a IE 6/178 Persistent BSI n/a Secondary localizations 43/178	[7]
Gazaigne L. (France, 2008)	Case series (1998-2006)	39 (9; 6; 24; 0)	67.6	18/21 (85.7%)	Median CCI n/a DM 5 CRF n/a CLD 3 CVD 2 PHV 1 IC 4	Inpatients (not specified)	n/a	n/a	CIP 4/21 ERY 1/21 TET 3/21 GEN 0/21 IPM 0/21	Fever 12 Sepsis n/a Septic shock 2 GI symptoms 4 Coinfections n/a	CIP (n°) 2 ERY (n°) 4 GEN (n°) 2 IPM (n°) 6 Other drugs (n°) 18 Combination therapy (n°) 11 N° days of therapy (median) 28 (9 days - 3.5 months)	5/18 (3 months)	Relapses 2/18 IE 1/18 Persistent BSI n/a Secondary localizations 12/18	[29]
Nielsen H. (Denmark, 2010)	Retrospective multicentric (1995-2004)	46 (37; 5; 3; 1)	50.3	33/46 (71.7%)	Median CCI n/a DM 3 CRF n/a CLD 4 CVD 11 PHV n/a IC 10	Inpatients (not specified)	6 abdominal surgery/trauma	n/a	CIP 9/35 ERY 3/40 TET n/a GEN 1/18 MEM n/a	Fever n/a Sepsis n/a Septic shock n/a GI symptoms 27 Coinfections n/a	Combination therapy (n°) 21	2/46 (28-day)	Relapses 0/46 IE 1/46 Persistent BSI n/a Secondary localizations 1/46	[30]
Fernández-Cruz A. (Spain, 2010)	Retrospective monocentric (1985-2007)	68 (45; 8; 13; 2)	51.4	56/68 (82.3%)	Median CCI n/a DM 0 CRF 3 CLD 21 CVD 3 PHV n/a IC 15	n/a	n/a	8	CIP 19/38 ERY 4/37 TET n/a GEN 2/35 IPM 0/30	Fever 55 Sepsis n/a Septic shock 9 GI symptoms 58 Coinfections 11	CIP (n°) 9 ERY (n°) 4 TET (n°) 1 GEN (n°) 1 IPM (n°) 2 Other drugs (n°) 27 Appropriate therapy (n°, %) 25/66 (38%)	11/68 (time point not specified)	Relapses 7/68 IE 0/68 Persistent BSI 0/68 Secondary localizations 0/68	[31]
Feodoroff B. (Finland, 2011)	Retrospective multicentric (1998-2007)	76 (73; 3; 0; 0)	47.3	56/76 (73.7%)	Median CCI n/a DM 1 CRF 1 CLD 4 CVD 1 PHV n/a IC 6	n/a	16 recent travel	n/a	CIP 5/76 ERY 0/76 TET 3/76 GEN 0/76 MEM 0/76	Fever 64 Sepsis n/a Septic shock n/a GI symptoms 60 Coinfections n/a	Appropriate therapy (n°, %) 50/76 (66%)	2/76 (30-day)	Relapses n/a IE 0/76 Persistent BSI 0/76 Secondary localizations 1/76	[32]
O’Hara G.A. (UK, 2017)	Retrospective monocentric (1972-2013)	41 (20; 3; 2; 16)	46	27/41 (65.8%)	Median CCI n/a DM 4 CRF 3 CLD 4 CVD n/a PHV n/a IC 12	Inpatients and outpatients (not specified)	n/a	n/a	CIP 9/26 ERY 1/30 TET n/a GEN n/a MEM n/a	Fever 35 Sepsis 1 Septic shock n/a GI symptoms 34 Coinfections n/a	CIP (n°) 5 Other drugs (n°) 16 Combination therapy (n°) 16 Appropriate therapy (n°, %) 7/29 (24%)	2/41 (time point not specified)	Relapses n/a IE 1/41 Persistent BSI n/a Secondary localization n/a	[33]
Tinévez C. (France, 2021)	Retrospective multicentric (2015-2019)	592 (254; 40; 252; 46)	66.3	402/592 (67.9%)	Median CCI n/a DM 128 CRF 118 CLD 75 CVD n/a PHV n/a IC 257	n/a	n/a	160	CIP 249/592 ERY 22/592 TET 167/592 GEN 3/592 IPM 0/592	Fever 426 Sepsis n/a Septic shock 43 GI symptoms 233 Coinfections 34	Combination therapy (n°) 133 N° days of therapy (median) 10 (5-15) N° days of therapy for complications (median) 41.5 (17-46) Appropriate therapy (n°, %) 430/551 (78%)	69/592 (30-day)	Relapses 18/592 IE 12/592 Persistent BSI n/a Secondary localizations 80/592	[8]
Graham A. (UK, 2024)	Retrospective multicentric (2012-2021)	34 (24; 2; 2; 6)	69.3	21/34 (61.7%)	Median CCI n/a DM 7 CRF n/a CLD n/a CVD 11 PHV n/a IC 10	n/a	n/a	27	CIP 14/28 ERY 0/28 TET n/a GEN n/a MEM n/a	Fever 12 Sepsis n/a Septic shock n/a GI symptoms 22 Coinfections n/a	GEN (n°) 6 MEM (n°) 13 Other drugs (n°) 23 N° days of therapy (median) 13 (10-14) Appropriate therapy (n°, %) 28/34 (82%)	1/34 (30-day)	Relapses n/a IE n/a Persistent BSI 1/34 Secondary localizations n/a	[34]
Sunnerhagen T. (Sweden, 2024)	Retrospective multicentric (2015-2022)	29 (11; 1; 2; 15)	70	22/29 (75.8%)	Median CCI 2 (0.5-5) DM 3 CRF 9 CLD 1 CVD 6 PHV n/a IC 15	Inpatients (not specified)	1 recent travel 2 contact with livestock/meat 2 outbreak involvement	6	CIP 6/18 ERY 1/18 TET n/a GEN n/a MEM n/a	Fever 24 Sepsis 6 Septic shock 2 GI symptoms 20 Coinfections n/a	CIP (n°) 5 ERY (n°) 4 Other drugs (n°) 12 N° days of therapy (median) 13 (9-17)	0/29 (30-day)	n/a	[35]
Liao C.-H. (Taiwan, 2012)	Case series (1998-2008)	24 (3; 15; 6; 0)	45.3	16/24 (66.7%)	Median CCI n/a DM 5 CRF 10 CLD 9 CVD 2 PHV n/a IC 16	n/a	8 abdominal surgery/trauma	1	CIP 15/24 ERY n/a TET n/a GEN n/a IPM 0/24	Fever 14 Sepsis n/a Septic shock n/a GI symptoms 15 Coinfections n/a	Other drugs (n°) 23 Appropriate therapy (n°, %) 11/24 (46%)	2/24 (30-day)	Relapses n/a IE n/a Persistent BSI 9/24 Secondary localizations 3/24	[36]
Liu Y. H. (Taiwan, 2019)	Retrospective monocentric (1998-2014)	56 (11; 26; 19; 0)	54	35/56 (62.5%)	Median CCI n/a DM 7 CRF 10 CLD 13 CVD 27 PHV n/a IC 30	n/a	n/a	n/a	n/a	Fever 10 Sepsis n/a Septic shock n/a GI symptoms 9 Coinfections n/a	n/a	3/56 (30-day)	Relapses n/a IE n/a Persistent BSI n/a Secondary localizations 1/56	[37]
Baek Y.J. (South Korea, 2023)	Retrospective multicentric (2010-2021)	108 (54; 7; 21; 26)	56	78/108 (72.2%)	Median CCI n/a DM 27 CRF 11 CLD 9 CVD 7 PHV n/a IC 31	n/a	n/a	n/a	CIP 45/76 ERY 3/73 TET 13/46 GEN 2/6 IPM 2/6	Fever 98 Sepsis n/a Septic shock 14 GI symptoms 65 Coinfections n/a	Other drugs (n°) 81 Appropriate therapy (n°, %) 27/105 (26%)	14/108 (in hospital)	Relapses n/a IE n/a Persistent BSI n/a Secondary localizations 31/108	[38]
Otsuka Y. (Japan, 2023)	Retrospective multicentric (2011-2021)	39 (27; 4; 5; 3)	53,2	24/39 (61.5%)	Median CCI n/a DM 8 CRF 4 CLD 5 CVD n/a PHV n/a IC 5	Inpatients and outpatients (not specified)	8 undecooked meat	n/a	n/a	Fever 35 Sepsis n/a Septic shock n/a GI symptoms 21 Coinfections n/a	N° days of therapy (median) 9 (4-15)	0/39 (in hospital)	Relapses n/a IE n/a Persistent BSI n/a Secondary localizations 6/39	[39]
Lastovica, AJ. (South Africa, 1996)	Retrospective monocentric (1977-1995)	238 (164; 4; 13; 57)	4.1	14/238 (5.9%)	Median CCI n/a DM n/a CRF n/a CLD 5 CVD 1 PHV n/a IC 2	n/a	2 abdominal surgery/trauma	n/a	n/a	Fever 6 Sepsis 58 Septic shock 0 GI symptoms 160 Coinfections n/a	n/a	n/a	n/a	[40]
Reed R.P. (South Africa, 1996)	Retrospective multicentric (1991-1994)	19 (19; 0; 0; 0)	1	11/19 (57.9%)	Median CCIn/a DM n/a CRF n/a CLD n/a CVD n/a PHV n/a IC 16	Medical ward	n/a	0	n/a	Fever 7 Sepsis n/a Septic shock n/a GI symptoms 15 Coinfections 1	Other drugs (n°) 11 N° days of therapy <= 5 days	3/19 (30-day)	n/a	[6]
Ben-Shimol S. (Israel, 2013)	Retrospective monocentric (1989-2010)	76 (27; 19; 0; 30)	4.7	55/76 (72.4%)	Median CCI n/a DM 0 CRF 2 CLD 2 CVD 0 PHV 0 IC 27	ED/medical ward	n/a	10	CIP 13/38 ERY 0/17 TET 13/30 GEN 0/25 MEM 0/7	Fever 47 Sepsis n/a Septic shock n/a GI symptoms 45 Coinfections n/a	n/a	0/76 (time point not specified)	n/a	[41]
Hussein K. (Israel, 2016)	Retrospective monocentric (2000-2015)	65 (33; 7; 5; 20)	42.3	36/55 (65.4%)	Median CCI n/a DM 8 CRF 7 CLD 9 CVD 9 PHV n/a IC 37	Inpatients (not specified)	n/a	4	CIP 38/58 ERY 2/59 TET 28/48 GEN 1/27 MEM 1/7	Fever 55 Sepsis n/a Septic shock n/a GI symptoms 34 Coinfections n/a	CIP 9 ERY 3 GEN 1 Other drugs (n°) 26 Combination therapy (n°) 16 N° days of therapy (median) 11 (2-23) Appropriate therapy (n°, %) 43/65 (66%)	3/65 (30-day)	Relapses 3/65 IE n/a Persistent BSI n/a Secondary localizations 6/65	[42]
Tau L. (Israel, 2022)	Retrospective monocentric (2007-2020)	76 (63; 11; 0; 2)	62.3	48/76 (63.1%)	Median CCI 5 (2-6) DM 19 CRF 15 CLD n/a CVD 29 PHV n/a IC 46	Inpatients (not specified)	n/a	14	CIP 55/70 ERY 2/69 TET n/a GEN n/a MEM n/a	Fever 64 Sepsis n/a Septic shock n/a GI symptoms 50 Coinfections n/a	Appropriate therapy (n°, %) 55/76 (72%)	9/76 (30-day)	Relapses 0/76 IE n/a Persistent BSI 0/76 Secondary localizations n/a	[43]
Morey, F. (Australia, 1996)	Retrospective monocentric (1990-1995)	72 (61; 0; 0; 11)	3	47/72 (65.3%)	Median CCI n/a DM n/a CRF n/a CLD n/a CVD n/a PHV n/a IC 13	n/a	n/a	n/a	n/a	n/a	n/a	n/a	n/a	[40]
Tee W. (Australia, 1998)	Retrospective monocentric (1985-1995)	21 (21; 0; 0; 0)	39.4	17/21 (81%)	Median CCI n/a DM n/a CRF n/a CLD n/a CVD n/a PHV n/a IC 12	n/a	5 recent travel	21	CIP 3/21 ERY 0/21 TET n/a GEN 0/21 MEM n/a	Fever 20 Sepsis 1 Septic shock n/a GI symptoms 18 Coinfections 6	CIP (n°) 10 ERY (n°) 9 TET (n°) 2 GEN (n°) 7 Other drugs (n°) 11 Combination therapy (n°) 15 Appropriate therapy (n°, %) 20/20 (100%)	3/21 (30-day)	Relapses 3/21 IE 0/21 Persistent BSI 1/21 Secondary localizations 4/21	[44]
Moffatt C.R.M. (Australia, 2021)	Case series (2004-2013)	25 (15; 5; 0; 5)	38.8	15/25 (60%)	Median CCI n/a DM 5 CRF 1 CLD 3 CVD n/a PHV n/a IC 8	Inpatients (not specified)	n/a	n/a	CIP 4/25 ERY 1/25 TET n/a GEN n/a MEM n/a	Fever n/a Sepsis n/a Septic shock n/a GI symptoms 23 Coinfections n/a	CIP (n°) 11 TET (n°) 1 Other drugs (n°) 3 Appropriate therapy (n°, %) 15/15 (100%)	0/25 (time point not specified)	n/a	[45]
Guerrant R.L. (USA, 1978)	Retrospective multicentric (n/a)	91 (10; 0; 50; 31)	n/a	65/91 (71.4%)	n/a	n/a	45 contact with livestock/cattle	n/a	n/a	Fever 84 Sepsis n/a Septic shock n/a GI symptoms 38 Coinfections n/a	n/a	18/91 (30-day)	n/a	[46]

**BSI** = Bloodstream infection; **CCI** = Charlson comorbidity index; **CIP** = ciprofloxacin; **CLD** = chronic liver disease; **CRF** = chronic renal failure; **CVD** = cardiovascular disease; **DM** = diabetes mellitus; **ERY** = erythromycin; **GEN** = gentamicin; **GI** = gastrointestinal; **IC** = immunocompromised; **IE** = infective endocarditis; **IPM** = imipenem; **MEM** = meropenem; **n/a** = not available; **PHV** = patients with prosthetic heart valves; **TET** = tetracycline; **UK** = United Kingdom; **USA** = United States of America. In the treatment column, we consider “Other drugs” to include all drugs except ciprofloxacin, erythromycin, tetracycline, gentamicin, meropenem, and imipenem.

**Table 2:** Univariate meta-regression of time trend in *Campylobacter* species proportions (worldwide).

Species	Coefficient	Standard Error (SE)	95% Confidence Interval (CI)	*p*-Value
*C. jejuni*	−0.007	0.024	−0.057;0.044	0.781
*C. coli*	0.046	0.024	−0.003;0.096	0.066
*C. fetus*	0.007	0.033	−0.059;0.074	0.825
Other species	−0.014	0.027	−0.070;0.041	0.593

**Table 3:** Results of univariate meta-regression analyses on mortality.

Variable	Coefficient	Standard Error (SE)	95% Confidence Interval (CI)	*p*-Value
Age (mean)	0.010	0.018	−0.028;0.047	0.589
Gender (male)	3.323	3.779	−4.609;11.254	0.390
Mid-year of the study	−0.015	0.023	−0.062;0.032	0.519
*C. jejuni*	−0.006	1.015	−2.137;2.124	0.995
*C. coli*	−1.024	1.787	−4.775;2.727	0.574
*C. fetus*	3.217	1.232	0.632;5.802	0.017
Other species	−3.808	1.760	−7.503; −0.114	0.044
Immunocompromised status	2.749	1.15	0.316;5.184	0.029
Chronic liver disease	5.072	2.173	0.424;9.720	0.034
Diabetes mellitus	2.521	3.421	−4.772;9.814	0.472
Chronic kidney disease	0.941	2.980	−5.494;7.377	0.757
Cardiovascular disease	0.241	2.327	−4.970;5.453	0.920
Concomitant positive stool culture	−0.691	1.117	−3.123;1.740	0.547
Ciprofloxacin resistance	1.140	1.574	−2.394;4.675	0.486
Tetracycline resistance	−1.146	1.966	−5.518;3.227	0.573
Erythromycin resistance	9.261	8.299	−9.115; 27.639	0.289
Gentamicin resistance	1.991	3.866	−6.613;10.596	0.617
Carbapenems resistance	1.070	3.114	−5.794;7.936	0.737
Days of therapy	−0.104	0.126	−0.464;0.255	0.457
Endocarditis	13.630	31.008	−56.644;83.904	0.670
Relapse	2.330	9.840	−21.155;25.814	0.820

**Table 4:** Results of multivariate meta-regression analyses on mortality.

Variable	Coefficient (Estimate)	Standard Error (SE)	95% CI	*p* Value
*C. fetus*	2.323	1.132	−0.125;4.771	0.061
*Campylobacter* other species	−3.134	1.564	−6.495;0.226	0.065
Immunocompromised status	1.738	1.089	−0.632;4.108	0.136
Chronic liver disease	0.710	2.382	−4.600;6.021	0.772

## Data Availability

No new data were created or analyzed in this study.

## Supplementary Materials

The following supporting information can be downloaded at: https://www.mdpi.com/article/10.3390/pathogens15070686/s1. PRISMA 2020 checklist; Rsearch Strategy; Supplementary Figures; Table S1; Other supplementary tables.
