# Supplementary material for: Clinical Outcomes of Campylobacter Bacteremia: A Systematic Review with Meta-Analysis
Source: Pathogens. 2026 Jun 29;15(7):686. doi: 10.3390/pathogens15070686 (PMC13414677; doi:10.3390/pathogens15070686)
Supplement: Supplementary file 1 [file pathogens-15-00686-s001.zip › Research strategy.pdf]

SEARCH STRATEGY

MEDLINE (PubMed) (inception to June 2024)

Campylobacter[Text Word] AND (bacteremia[Text Word] OR bacteraemia[Text Word] OR BSI[Text Word] OR bloodstream[Text Word])

Embase (Embase.com) (inception to June 2024)

campylobacter:ti,ab,kw AND (bacteremia:ti,ab,kw OR bacteraemia:ti,ab,kw OR BSI:ti,ab,kw OR bloodstream:ti,ab,kw)

Web of Science (Clarivate.com) (inception to June 2024)

Campylobacter AND (bacteremia OR bacteraemia OR BSI OR bloodstream)
