# Supplementary material for: Clinical Outcomes of Campylobacter Bacteremia: A Systematic Review with Meta-Analysis
Source: Pathogens. 2026 Jun 29;15(7):686. doi: 10.3390/pathogens15070686 (PMC13414677; doi:10.3390/pathogens15070686)

Figure S1: Forest plot illustrating the pooled prevalence of strains resistant to ciprofloxacin.

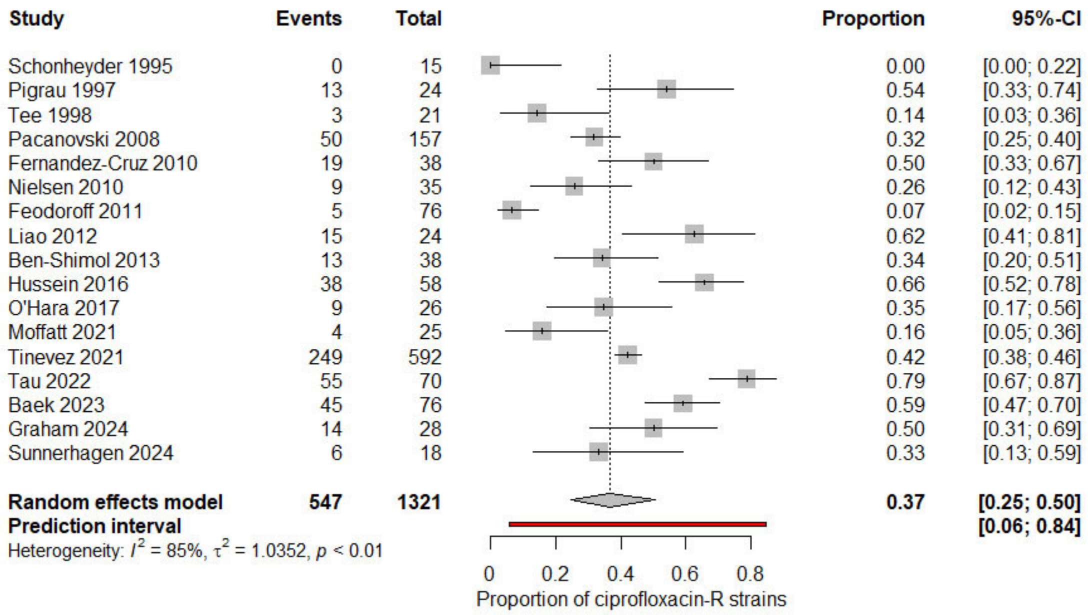

Figure S2: Forest plot illustrating the pooled prevalence of strains resistant to erythromycin.

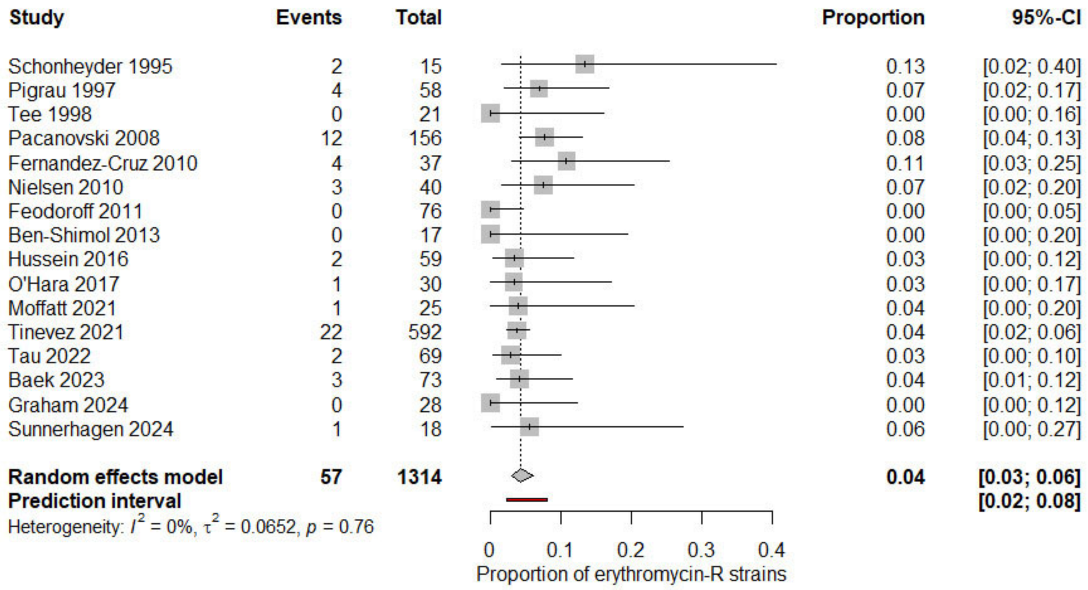

Figure S3: Forest plot illustrating the pooled prevalence of strains resistant to tetracycline.

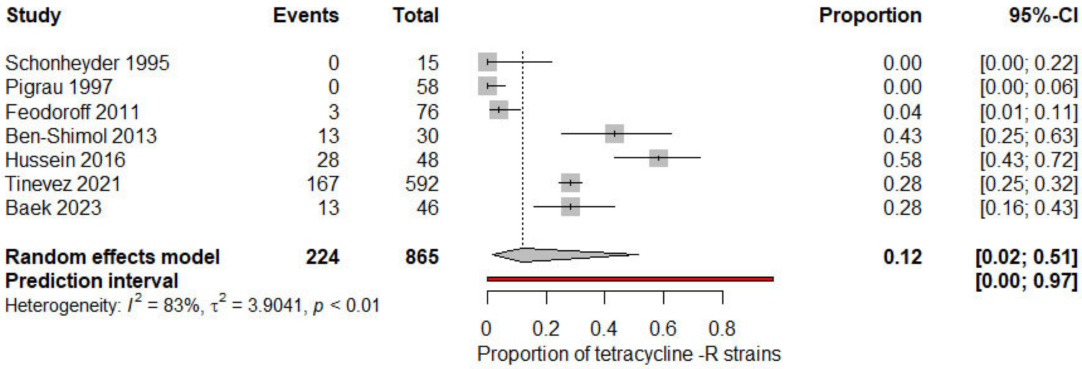

Figure S4: Forest plot illustrating the pooled prevalence of strains resistant to gentamicin.

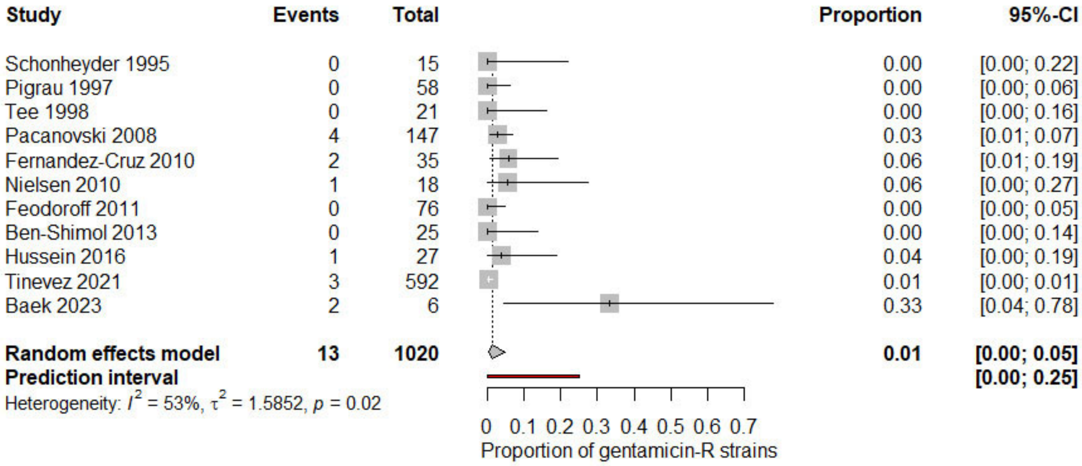

Figure S5: Forest plot illustrating the pooled prevalence of strains resistant to carbapenems.

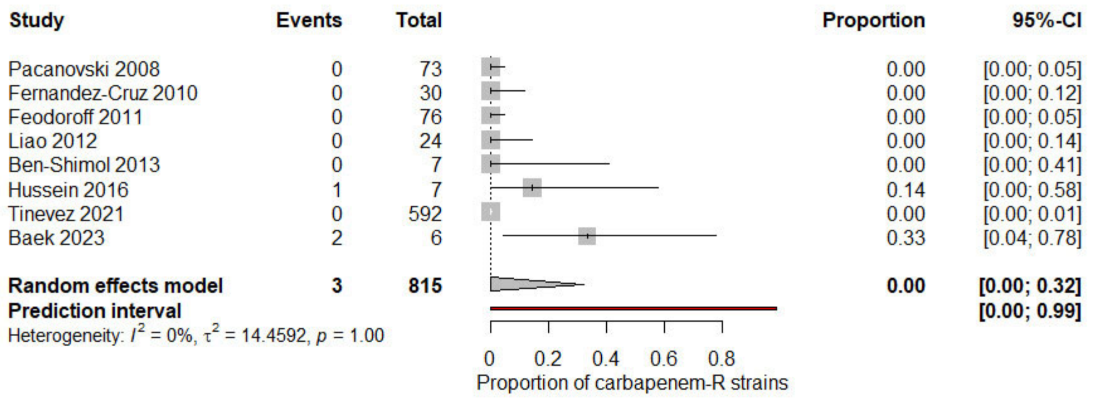

Figure S6: Forest plot illustrating the pooled prevalence of fever at the time of bloodstream infection (BSI) diagnosis.

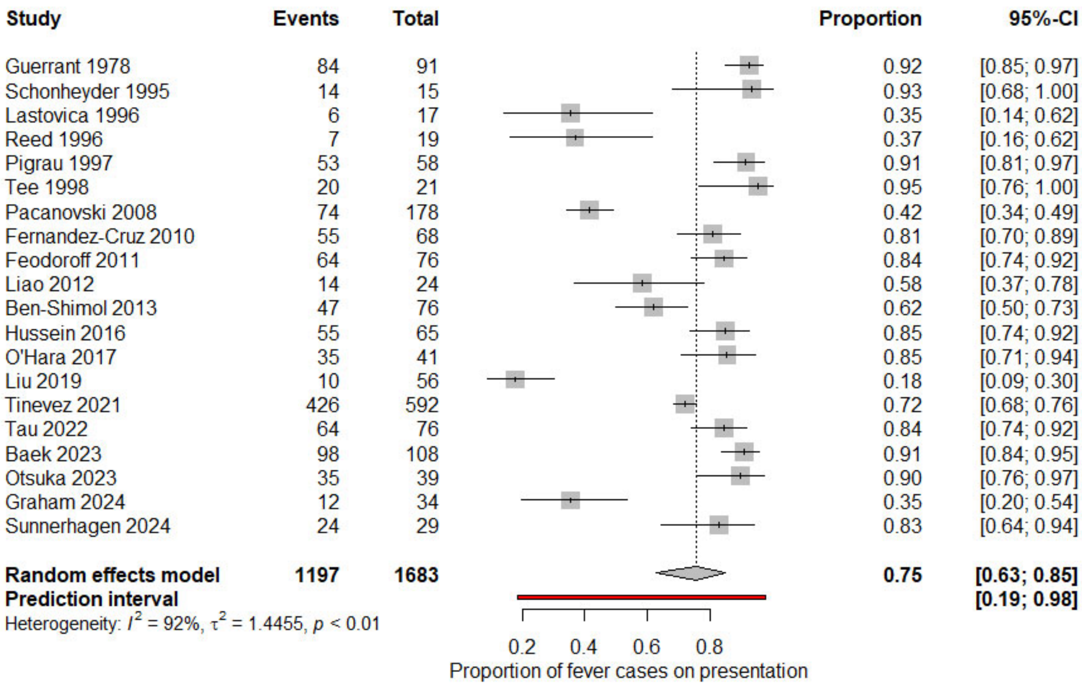

Figure S7: Forest plot illustrating the pooled prevalence of gastrointestinal manifestations at the time of bloodstream infection (BSI) diagnosis.

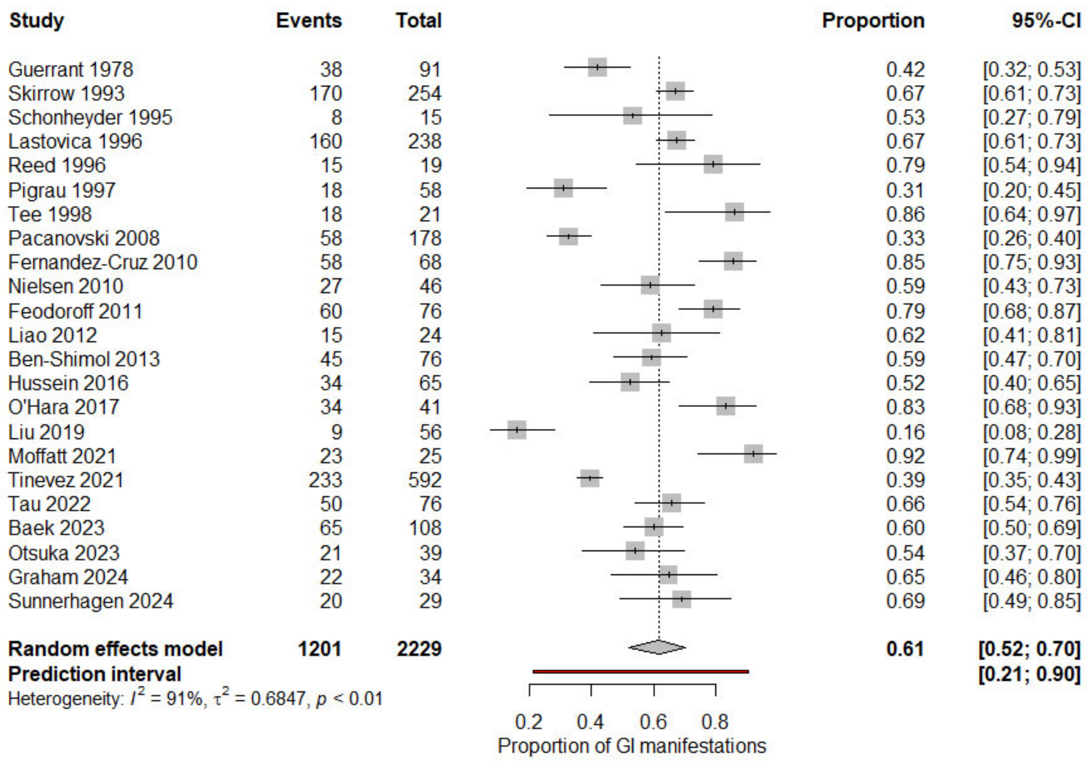

Figure S8: Forest plot illustrating the pooled prevalence of concomitant positive stool cultures at the time of bloodstream infection (BSI) diagnosis.

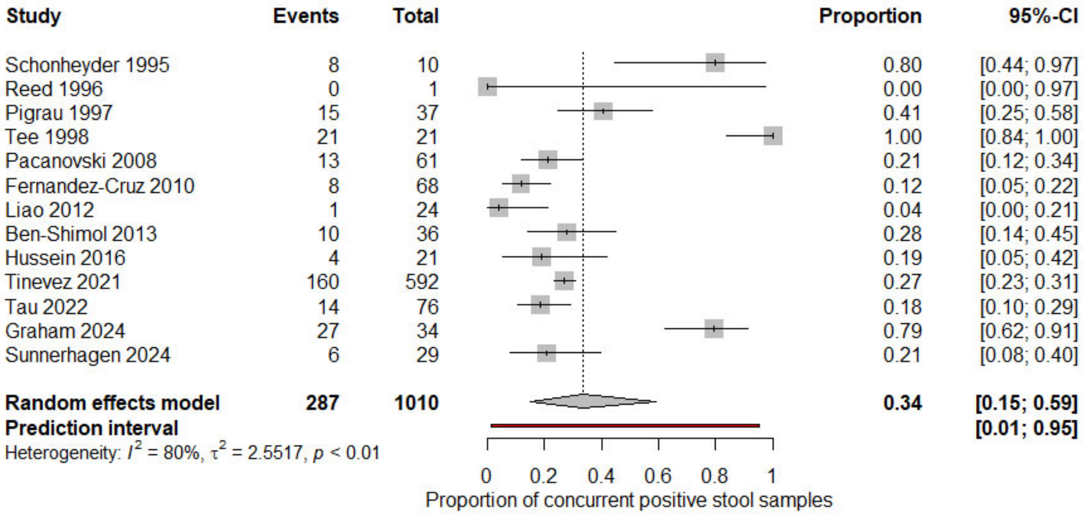

Figure S9: Forest plot illustrating the pooled prevalence of secondary localizations.

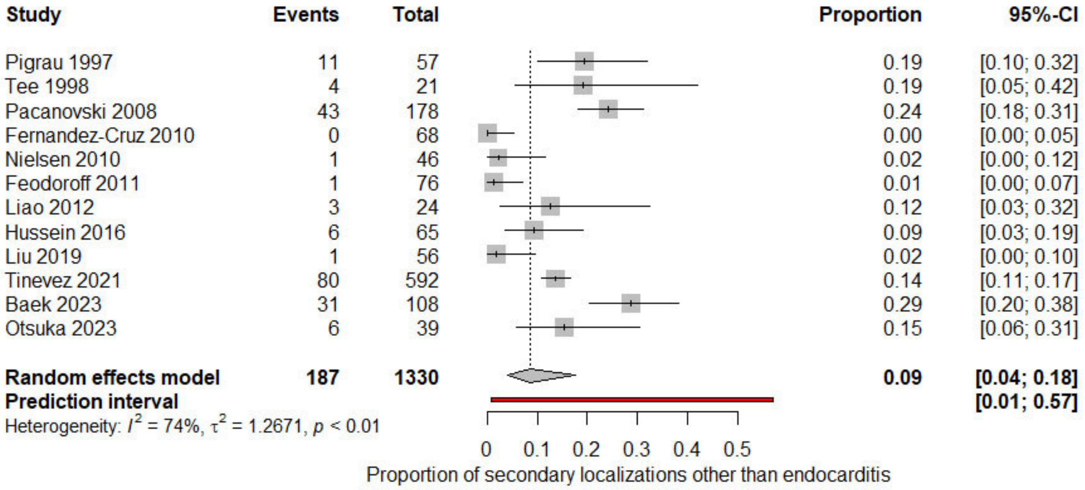

Figure S10: Forest plot illustrating the pooled prevalence of infective endocarditis.

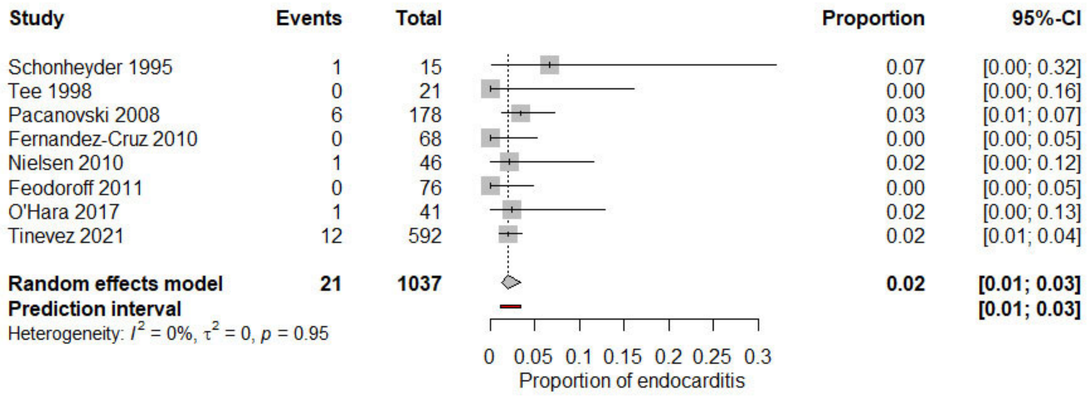

Figure S11: Forest plot illustrating the pooled prevalence of relapses.

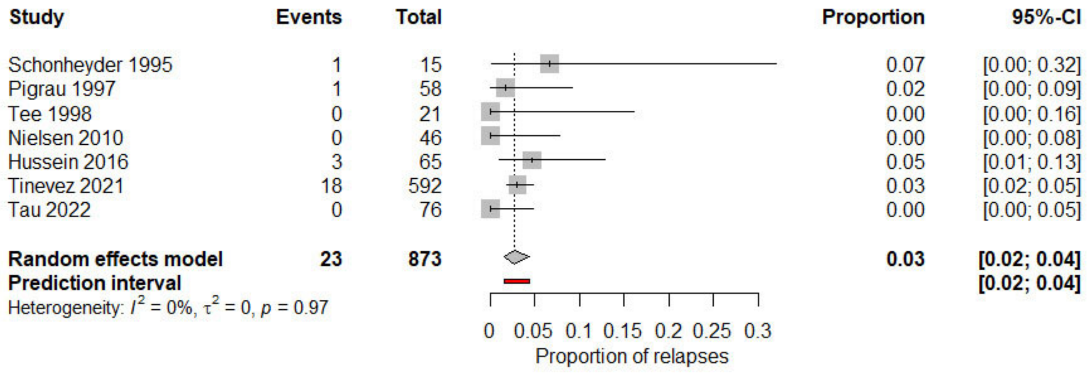

Figure S12: Forest plot illustrating the pooled prevalence of persistent cases of BSI.

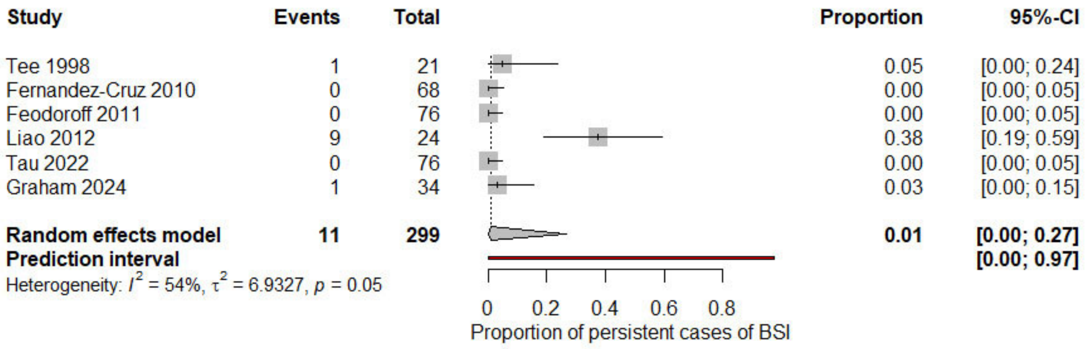

Figure S13: Forest plot showing the risk difference in adverse outcomes between appropriate and inappropriate therapy.

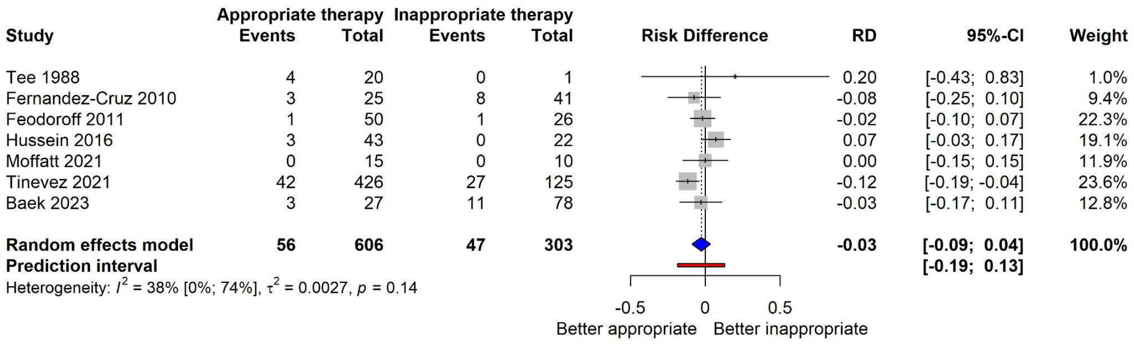

Figure S14: Meta-regression analysis of ciprofloxacin resistance in *Campylobacter* over time.

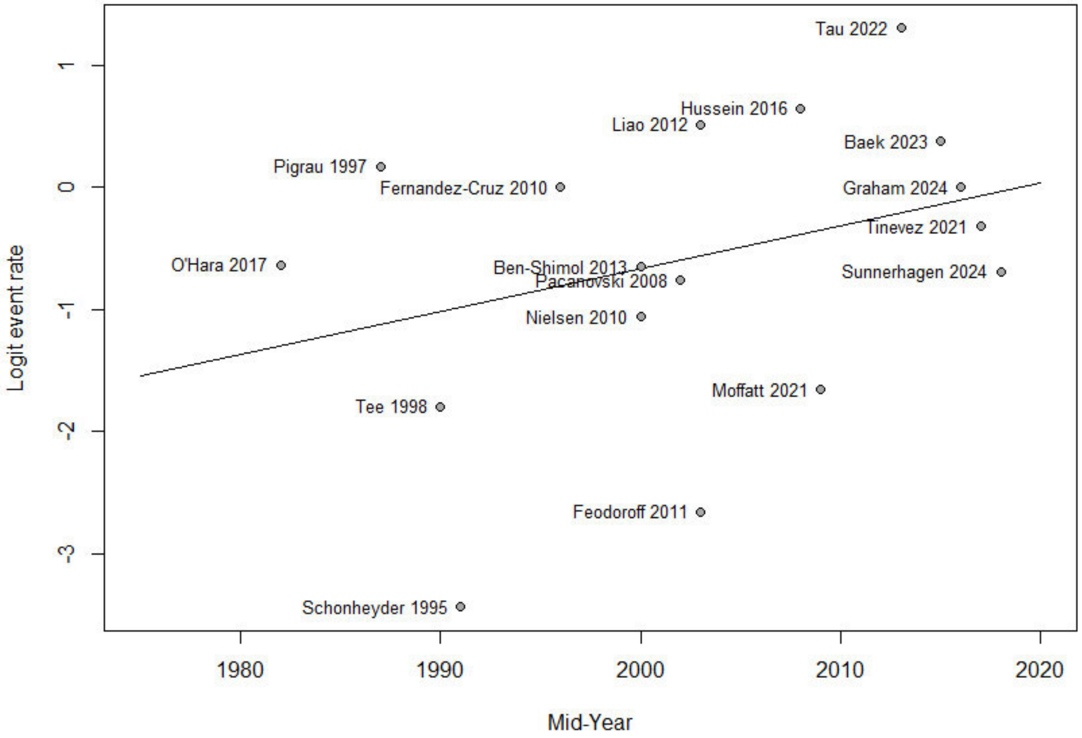

Figure S15: Meta-regression analysis of tetracycline resistance in *Campylobacter* over time.

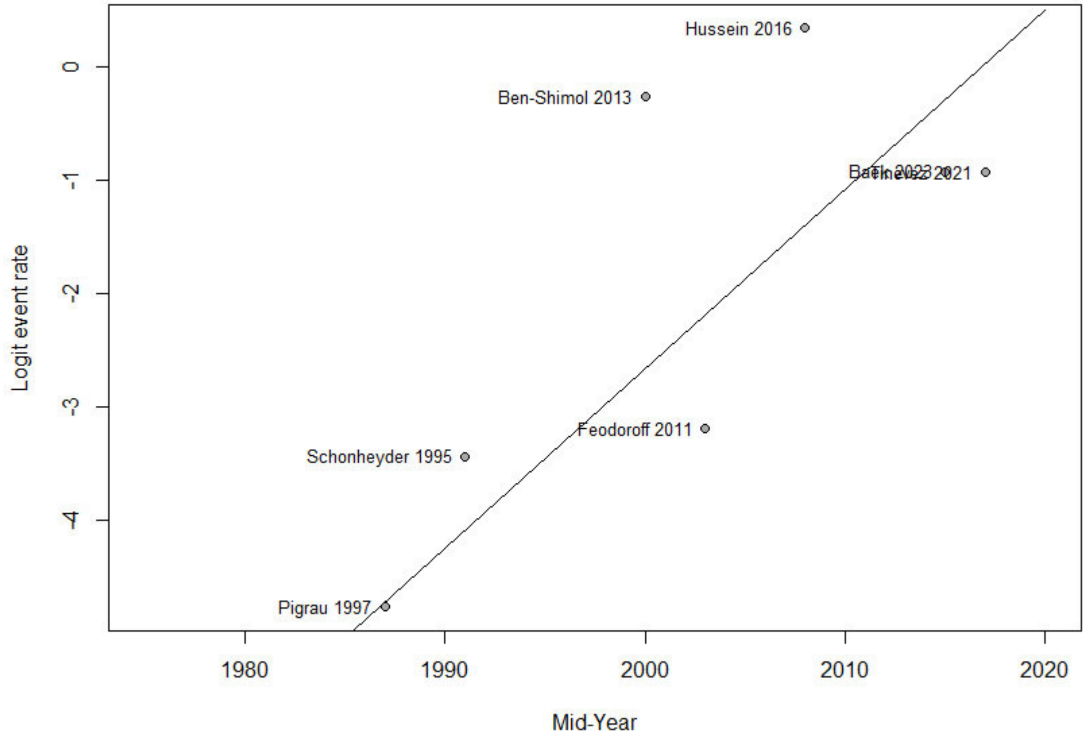

Figure S16: Doi Plot regarding proportion of *C. jejuni*.

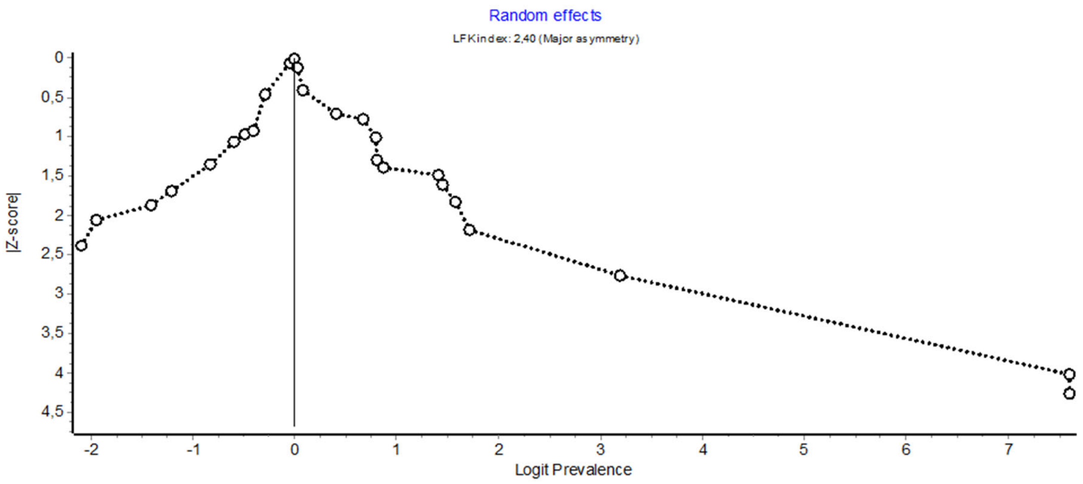

Figure S17: Doi Plot regarding Mortality.

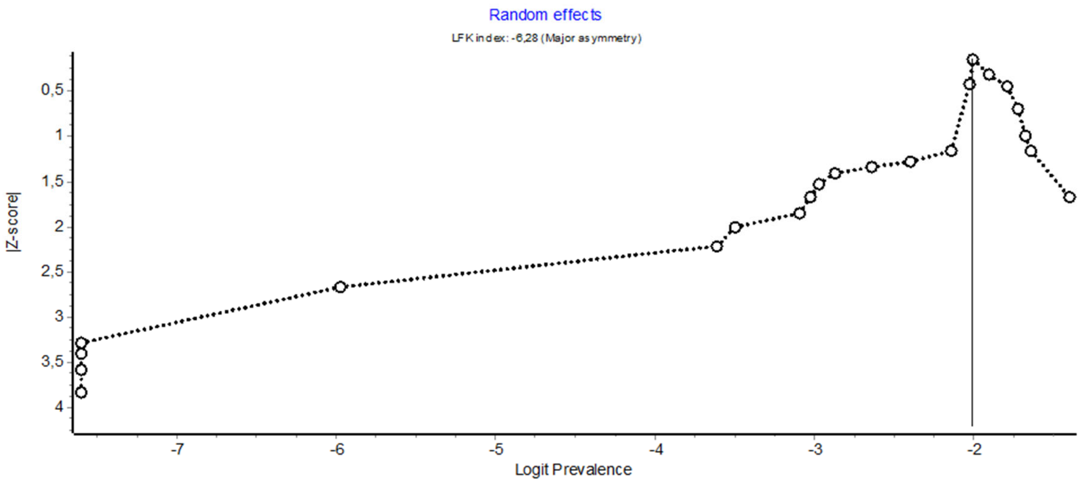

Figure S18: Forest plot illustrating the pooled analysis of mortality outcome according WHO region.

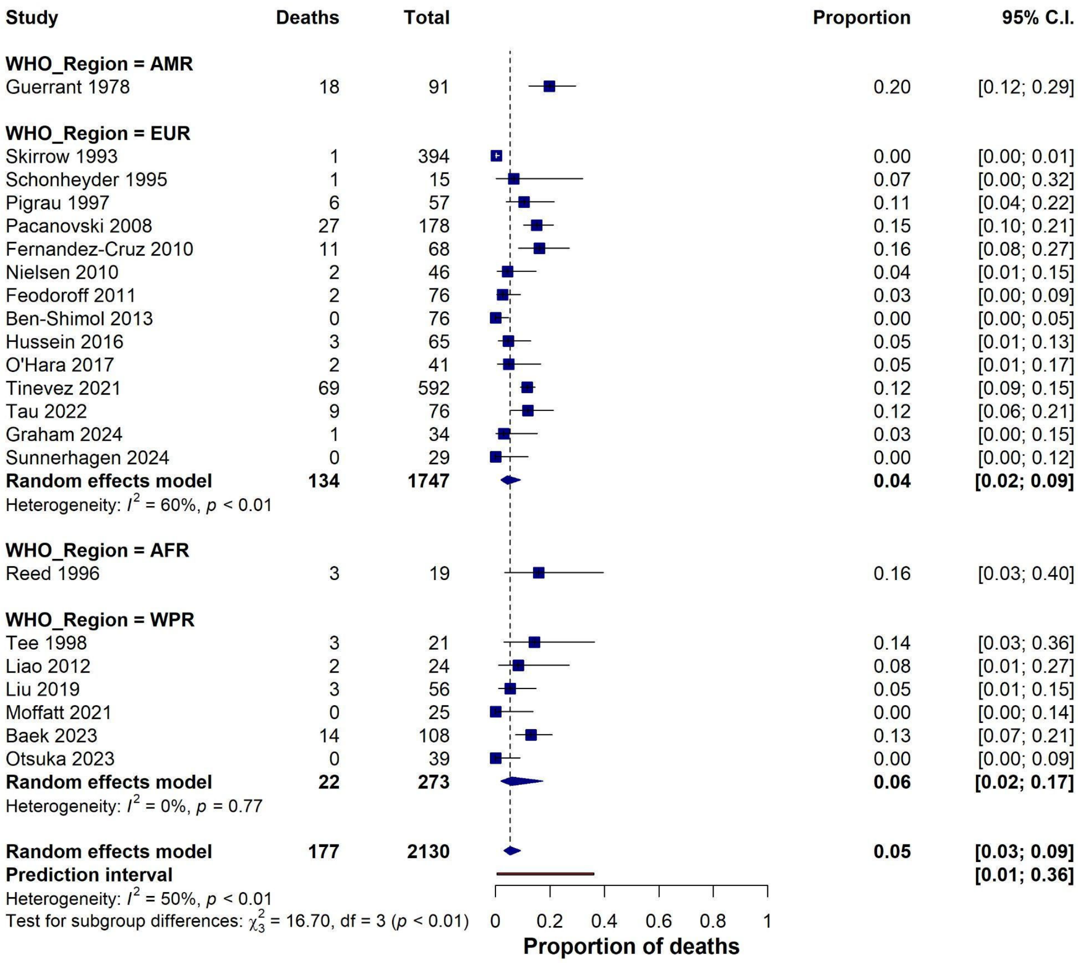

Figure S19: Missing pattern across studies.
Blue cells indicate available data; pink cells indicate missing values. Columns represent variables, rows represent individual studies.

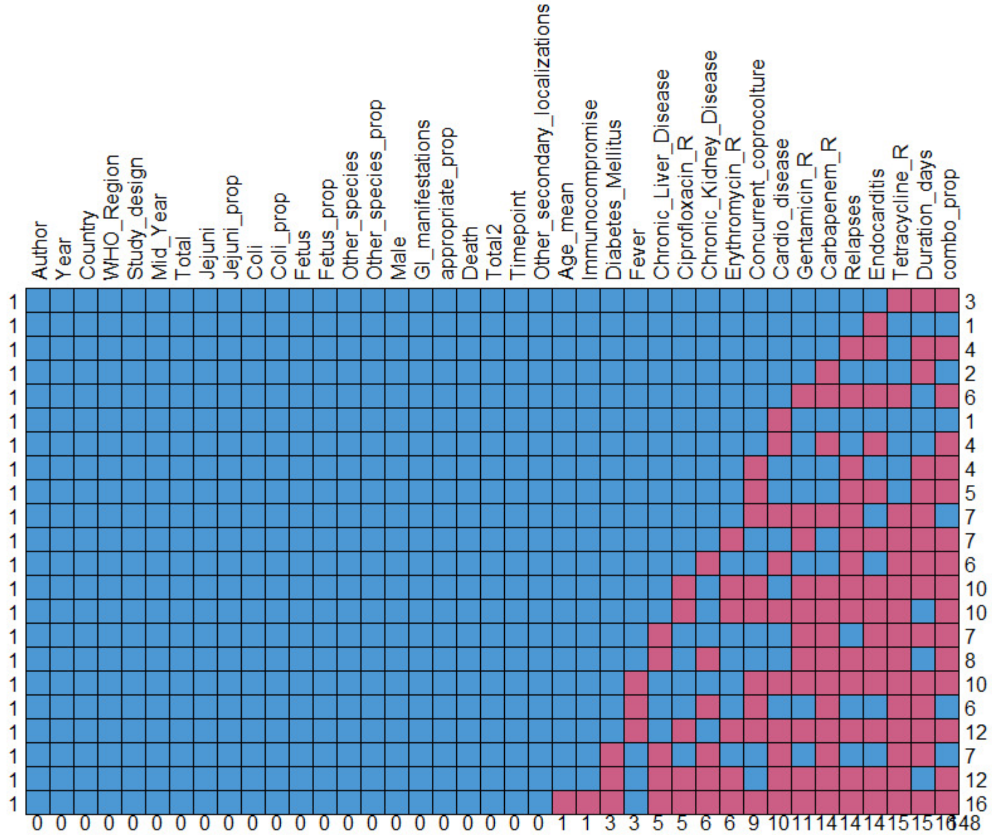

Figure S20: Influence analysis of mortality.

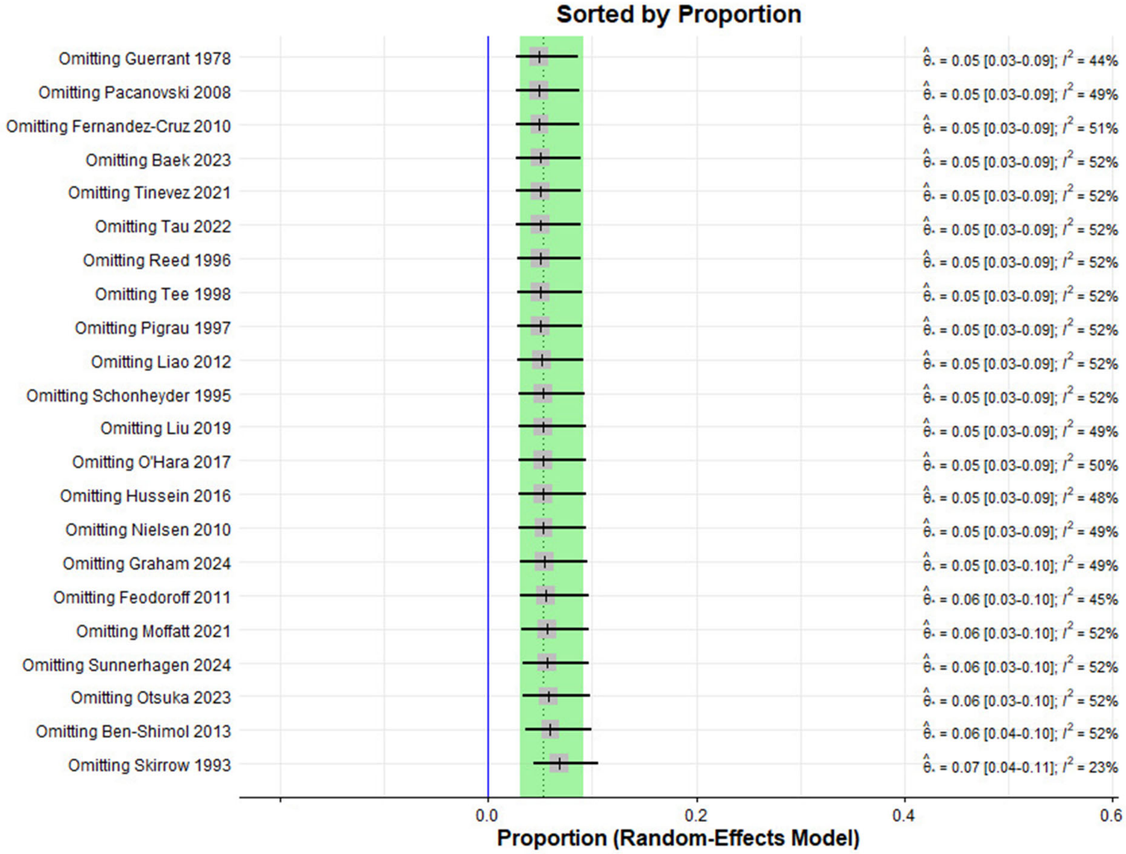

Figure S21: Influence analysis of the pooled odds ratio for appropriate treatment.

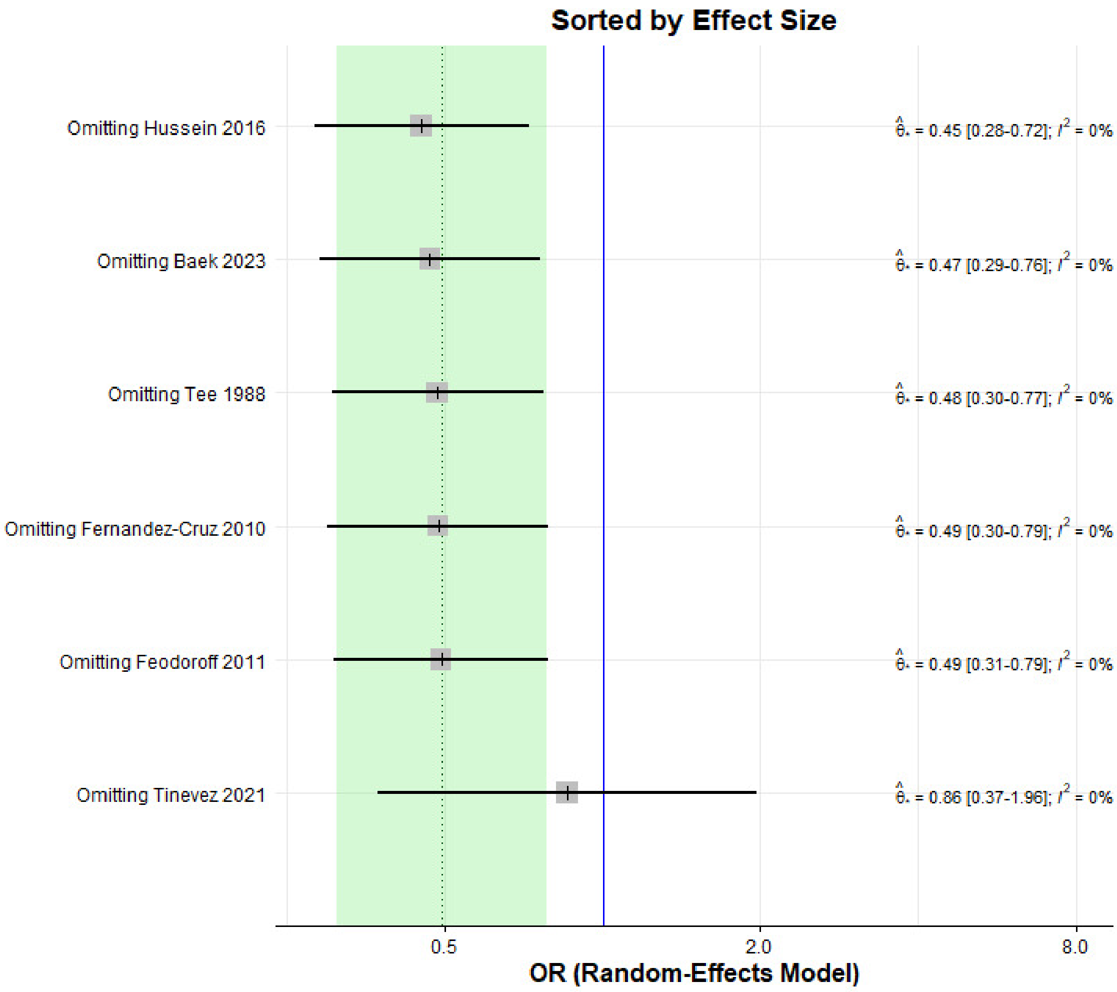

Figure S22: Influence analysis of the pooled risk difference for appropriate treatment.

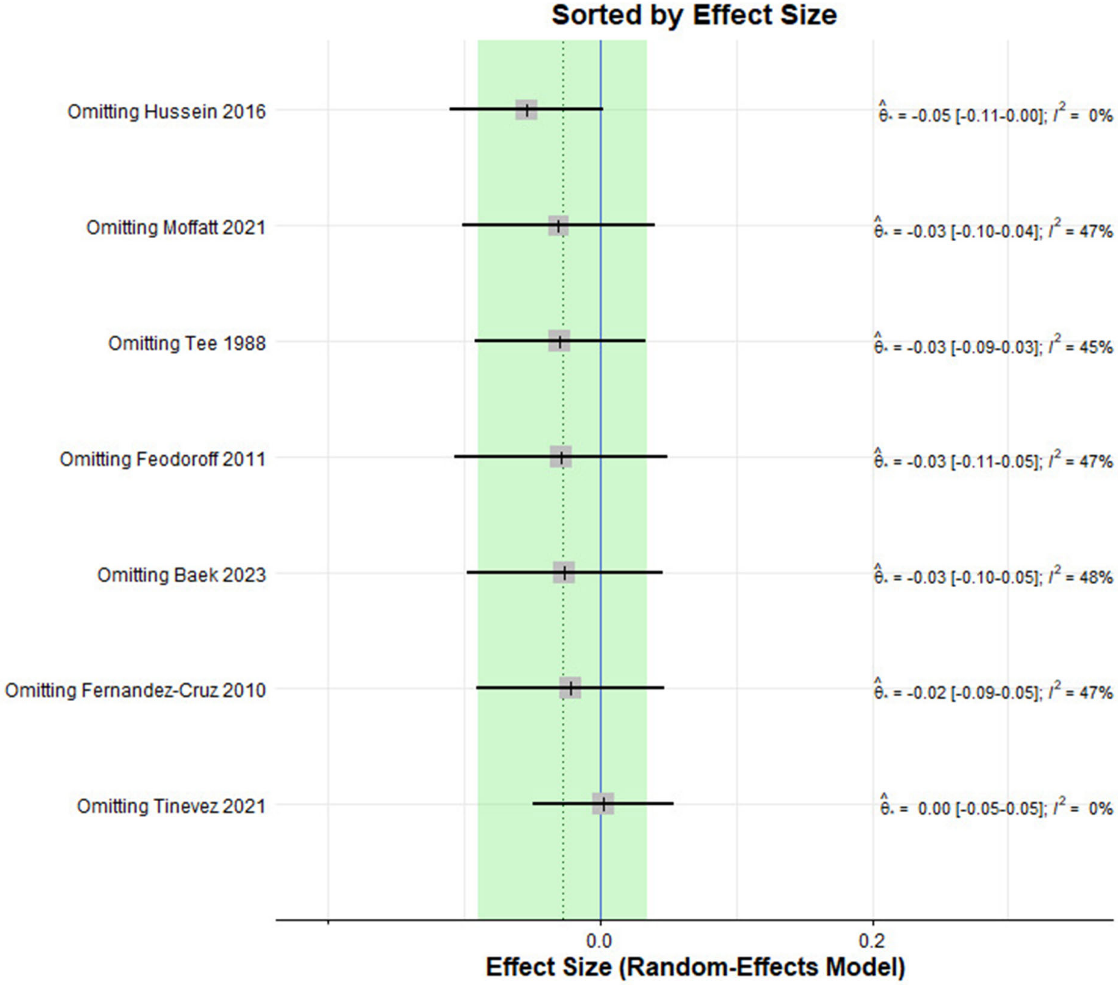

Supplement: Supplementary file 1 [file pathogens-15-00686-s001.zip › Supplementary Figures.pdf]
