# Supplementary material for: Clinical Outcomes of Campylobacter Bacteremia: A Systematic Review with Meta-Analysis
Source: Pathogens. 2026 Jun 29;15(7):686. doi: 10.3390/pathogens15070686 (PMC13414677; doi:10.3390/pathogens15070686)
Supplement: Supplementary file 1 [file pathogens-15-00686-s001.zip › Table S1.pdf]

Table S1. Evaluation of bias of included studies (with regard to Campylobacter strains proportion) based on the Hoy's criteria.

Reference	*Q1*	*Q2*	*Q3*	*Q4*	*Q5*	*Q6*	*Q7*	*Q8*	*Q9*	*Q10*	*Overall score*
**Skirrow M.B. (UK 1993)**	1	1	1	1	1	0	0	1	1	1	**8**
**Schønheyder H.C. (Denmark 1995)**	1	1	1	1	1	1	0	1	1	1	**9**
**Pigrau C. (Spain 1997)**	0	1	1	1	1	1	1	1	1	1	**9**
**Pacanowski J. (France 2008)**	1	1	1	1	1	1	0	1	1	1	**9**
**Gazaigne L. (France 2008)**	0	1	1	1	1	1	1	1	1	1	**9**
**Nielsen H. (Denmark 2010)**	1	1	1	1	1	1	0	1	1	1	**9**
**Fernández-Cruz A. (Spain 2010)**	0	1	1	1	1	1	0	1	1	1	**8**
**Feodoroff B. (Finland 2011)**	1	1	1	1	1	0	0	1	1	1	**8**
**O'Hara G.A. (UK 2017)**	0	1	1	1	1	1	0	1	1	1	**8**
**Tinévez C. (France 2021)**	1	1	1	1	1	1	1	1	1	1	**10**
**Graham A. (UK 2024)**	1	1	1	1	1	1	1	1	1	1	**10**
**Sunnerhagen T. (Sweden 2024)**	1	1	1	1	1	1	0	1	1	1	**9**
**Liao C.-H. (Taiwan 2012)**	0	1	1	1	1	1	1	1	1	1	**9**
**Liu Y.H. (Taiwan 2019)**	0	1	1	1	1	1	0	1	1	1	**8**

Reference	*Q1*	*Q2*	*Q3*	*Q4*	*Q5*	*Q6*	*Q7*	*Q8*	*Q9*	*Q10*	*Overall score*
**Baek Y.J. (South Korea 2023)**	0	1	1	1	1	1	0	1	1	1	**8**
**Otsuka Y. (Japan 2023)**	1	1	1	1	1	1	0	1	1	1	**9**
**Lastovica A.J. (South Africa 1996)**	0	1	1	1	1	1	0	1	1	0	**7**
**Reed M.B. (South Africa 1996)**	0	1	1	1	1	1	1	1	1	1	**9**
**Ben-Shimol S. (Israel 2013)**	0	1	1	1	1	1	1	1	1	1	**9**
**Hussein K. (Israel 2016)**	0	1	1	1	1	1	1	1	1	1	**9**
**Tau L. (Israel 2022)**	0	1	1	1	1	1	0	1	1	1	**8**
**Morey F. (Australia 1996)**	0	1	1	1	1	1	0	1	1	0	**7**
**Tee W. (Australia 1998)**	0	1	1	1	1	1	1	1	1	1	**9**
**Moffatt C.R.M. (Australia 2021)**	0	1	1	1	1	0	0	1	1	1	**7**
**Guerrant R.L. (USA 1978)**	1	1	1	1	1	1	1	0	1	1	**9**

Quality assessment of included studies was carried out through the tool developed by Hoy and colleagues [1]. A score of 1 (yes) or 0 (no) was attributed for each item, ranging the final quality score from 0 to 10. Studies were therefore labelled as having a low (> 8), moderate (6–8), or high (≤ 5) risk of bias.

[1]: Hoy D, Brooks P, Woolf A, et al. Assessing risk of bias in prevalence studies: modification of an existing tool and evidence of interrater agreement. J Clin Epidemiol 2012; 65 (9): 934-9.

List of 10 questions (Q1 – Q10) applied to the included studies adapted to the purposes of the present review:

Here is a modified version of the 10 questions, adapted to assess studies on the prevalence of the causative strains of **Campylobacter bacteremia**, focusing on external and internal validity for inclusion in a meta-analysis of proportions:

External Validity

1. **Was the study's target population a close representation of the national or relevant population in relation to key variables for Campylobacter bacteremia?**
   - Assess whether the study population reflects the broader population affected by Campylobacter bacteremia in terms of relevant variables (e.g., age, geography, healthcare access).

2. **Was the sampling frame a true or close representation of the population at risk of Campylobacter bacteremia?**
   - Evaluate whether the sampling frame from which cases were drawn accurately reflects the population at risk for Campylobacter bacteremia, including hospitalized patients, immunocompromised individuals, etc.

3. **Was a consecutive sample of Campylobacter bacteremia cases undertaken or some form of random selection used to make the sample?**
   - Consider whether the sample was selected randomly or systematically (e.g., consecutive cases).

4. **Was the likelihood of nonresponse or selection bias minimal in the identification of Campylobacter bacteremia cases?**
   - Determine if certain groups (e.g., asymptomatic carriers, mild cases) were systematically excluded from the study, potentially introducing bias into the reported prevalence.

Internal Validity

5. **Were data on Campylobacter bacteremia collected directly from confirmed clinical cases (as opposed to proxy reports or assumptions)?**

- Assess whether the data on Campylobacter strains came from laboratory-confirmed cases of bacteremia rather than indirect sources or proxy reporting.

6. **Was the diagnostic method or study instrument for identifying the Campylobacter strains (e.g., species identification, serotyping, or molecular methods) shown to have validity and reliability?**

- Check whether the diagnostic methods used to identify Campylobacter strains (e.g., PCR, MALDI-TOF, culture methods) were validated and reliable in the context of bacteremia studies.

7. **Was the same mode of data collection (e.g., diagnostic testing) used for all subjects in the study?**

- Determine whether all subjects were tested using the same diagnostic approach to ensure consistency in strain identification across the sample.

8. **Was the length of the study period appropriate for capturing the prevalence of Campylobacter bacteremia and its causative strains (at least 5 years)?**

- Evaluate whether the study duration was sufficient to capture seasonal and temporal variations in Campylobacter bacteremia cases and the strains causing the infection.

9. **Did the sample have an adequate sample size (at least 15 cases)?**

- Assess whether the study clearly defined and appropriately calculated the numerator (cases of specific Campylobacter strains) and the denominator (total cases of Campylobacter bacteremia or the population at risk).

10. **Was mortality as consequence of Campylobacter bacteremia reported in the study as outcome?**

- Ensure that the study used collected data on mortality.
