# Supplementary material for: Clinical Outcomes of Campylobacter Bacteremia: A Systematic Review with Meta-Analysis
Source: Pathogens. 2026 Jun 29;15(7):686. doi: 10.3390/pathogens15070686 (PMC13414677; doi:10.3390/pathogens15070686)
Supplement: Supplementary file 1 [file pathogens-15-00686-s001.zip › Other supplementary tables.pdf]

Table S2: Univariate meta-regression results for *C. jejuni* time trends by WHO region.

**Region**	**Coefficient**	**Standard error (SE)**	**95% Confidence Interval**	**P-value**
Europe<br><br>(15 studies)	-0.0002	0.024	-0.054;0.053	0.993
Africa* (2 studies)	4.151	8847.3019	NA	NA
Western Pacific (7 studies)	-0.100	0.066	-0.271;0.070	0.191
America (1 study)	no meta-regression			

**Fallback to fixed effect model due to small number of studies.*

Table S3: Univariate meta-regression results for *C. coli* time trends by WHO region.

**Region**	**Coefficient**	**Standard error (SE)**	**95% Confidence Interval**	**P-value**
Europe<br><br>(15 studies)	-0.002	0.017	-0.039;0.035	0.909
Africa* (2 studies)	-3.605	8847.2900	NA	NA
Western Pacific (7 studies)	0.142	0.109	-0.138;0.423	0.249
America (1 study)	no meta-regression			

**Fallback to fixed effect model due to small number of studies.*

Table S4: Univariate meta-regression results for *C. fetus* time trends by WHO region.

**Region**	**Coefficient**	**Standard error (SE)**	**95% Confidence Interval**	**P-value**

Europe<br><br>(15 studies)	0.005	0.042	-0.085;0.096	0.903
Africa* (2 studies)	-3.808	8847.2900	NA	NA
Western Pacific (7 studies)	0.155	0.096	-0.093;0.403	0.169
America (1 study)	no meta-regression			

**Fallback to fixed effect model due to small number of studies.*

Table S5: Univariate meta-regression results for other *Campylobacter* species time trends by WHO region.

**Region**	**Coefficient**	**Standard error (SE)**	**95% Confidence Interval**	**P-value**
Europe<br><br>(15 studies)	-0.011	0.036	-0.089;0.066	0.759
Africa* (2 studies)	-4.091	8847.3019	NA	NA
Western Pacific (7 studies)	0.061	0.072	-0.123;0.246	0.434
America (1 study)	no meta-regression			

**Fallback to fixed effect model due to small number of studies.*

Table S6: Meta-regression analysis of temporal trends in ciprofloxacin and tetracycline resistance among *Campylobacter* isolates.

**Antibiotic**	**Coefficient**	**Standard Error (SE)**	**95% Confidence Interval**	**P-value**
Ciprofloxacin	0.016	0.073	-0.030;0.3461	0.084
Tetracycline	0.035	0.024	-0.015;0.0852	0.158
